# Insights into early evolutionary adaptations of the *Akkermansia* genus to the vertebrate gut

**DOI:** 10.3389/fmicb.2023.1238580

**Published:** 2023-09-14

**Authors:** Dámariz González, Mauricio Morales-Olavarria, Boris Vidal-Veuthey, Juan P. Cárdenas

**Affiliations:** ^1^Centro de Genómica y Bioinformática, Facultad de Ciencias, Ingeniería y Tecnología, Universidad Mayor, Santiago, Chile; ^2^Escuela de Biotecnología, Facultad de Ciencias, Ingeniería y Tecnología, Universidad Mayor, Santiago, Chile

**Keywords:** *Akkermansia*, pangenome, phylogenomics, gene gain/loss model, dN/dS, Tajima D value, mucin degradation

## Abstract

*Akkermansia*, a relevant mucin degrader from the vertebrate gut microbiota, is a member of the deeply branched Verrucomicrobiota, as well as the only known member of this phylum to be described as inhabitants of the gut. Only a few *Akkermansia* species have been officially described so far, although there is genomic evidence addressing the existence of more species-level variants for this genus. This niche specialization makes *Akkermansia* an interesting model for studying the evolution of microorganisms to their adaptation to the gastrointestinal tract environment, including which kind of functions were gained when the *Akkermansia* genus originated or how the evolutionary pressure functions over those genes. In order to gain more insight into *Akkermansia* adaptations to the gastrointestinal tract niche, we performed a phylogenomic analysis of 367 high-quality *Akkermansia* isolates and metagenome-assembled genomes, in addition to other members of Verrucomicrobiota. This work was focused on three aspects: the definition of *Akkermansia* genomic species clusters and the calculation and functional characterization of the pangenome for the most represented species; the evolutionary relationship between *Akkermansia* and their closest relatives from Verrucomicrobiota, defining the gene families which were gained or lost during the emergence of the last *Akkermansia* common ancestor (*LAkkCA*) and; the evaluation of the evolutionary pressure metrics for each relevant gene family of main *Akkermansia* species. This analysis found 25 *Akkermansia* genomic species clusters distributed in two main clades, divergent from their non-*Akkermansia* relatives. Pangenome analyses suggest that *Akkermansia* species have open pangenomes, and the gene gain/loss model indicates that genes associated with mucin degradation (both glycoside hydrolases and peptidases), (micro)aerobic metabolism, surface interaction, and adhesion were part of *LAkkCA*. Specifically, mucin degradation is a very ancestral innovation involved in the origin of *Akkermansia*. Horizontal gene transfer detection suggests that *Akkermansia* could receive genes mostly from unknown sources or from other Gram-negative gut bacteria. Evolutionary metrics suggest that *Akkemansia* species evolved differently, and even some conserved genes suffered different evolutionary pressures among clades. These results suggest a complex evolutionary landscape of the genus and indicate that mucin degradation could be an essential feature in *Akkermansia* evolution as a symbiotic species.

## 1. Introduction

*Akkermansia* is a genus of anaerobic mucin-degrading Gram-negative bacteria from the Verrucomicrobiota phylum (Derrien et al., 2004). *Akkermansia muciniphila*, the first characterized member of this genus, was isolated from human stool with a medium containing mucin as the primary carbon and nitrogen source (Derrien et al., 2004). The second species described in the genus was *A. glycaniphila*, isolated from reticulated python feces (Ouwerkerk et al., 2016a), and from human stool (Lv et al., 2020). More recently, species such as *A. biwaensis*, *A. massiliensis*, *Candidatus* Akkermansia intestinavium, *Ca.* Akkermansia intestinigallinarum and *Ca.* Akkermansia timonensis, have been also proposed (Ndongo et al., 2022). According to these findings, other analyses from multiple metagenomic datasets suggest that there are numerous species related to *A. muciniphila*, and some of them coexist in different samples from human microbiomes (van Passel et al., 2011; Guo et al., 2017; Xing et al., 2019; Lv et al., 2020; Karcher et al., 2021). In addition, *Akkermansia*-like microorganisms have been detected in samples from other different vertebrates, including primates [e.g., lemurs, gorillas (Ley et al., 2008)] or mice (Presley et al., 2010), several orders of mammals (Geerlings et al., 2021), as well as chickens (Belzer and de Vos, 2012) or reptiles (Costello et al., 2010; Ouwerkerk et al., 2016a).

In the human gastrointestinal (GI) tract microbiome, *A. muciniphila* represents 1–4% of the microbial composition, detectable in a considerable fraction of reference populations (Belzer and de Vos, 2012). Due to its mucin-degrading role, this microbe plays a positive role in maintaining a healthy mucous layer and gut barrier integrity (Derrien et al., 2017). In addition, *A. muciniphila* levels were observed to decrease in inflammatory bowel disease (Png et al., 2010). Furthermore, *Akkermansia* abundance also decreases in subjects with metabolic disorders such as obesity and diabetes, as seen in studies with both murine models and human cohorts [reviewed in Derrien et al. (2017)]. Moreover, intervention studies in mice (Everard et al., 2013) and proof-of-concept studies in human subjects (Depommier et al., 2019) showed that *A. muciniphila* intake could improve parameters associated with insulin resistance, such as glycemia or insulin sensibility, among other factors. These findings showed the potential of *A. muciniphila* as a new probiotic agent and a source of postbiotics (Vinderola et al., 2022).

From an evolutionary perspective, *Akkermansia* is an interesting case of a member of the GI microbiota with close relatives inhabiting non-related environments. Furthermore, *Akkermansia*-related species are almost the only members of the Verrucomicrobiota phylum identified in the vertebrate gut microbiome, especially in mammals (Youngblut et al., 2020; Levin et al., 2021). The members of the Verrucomicrobiota (formerly Verrucomicrobia) phylum (Hedlund, 2015) are Gram-negatives with additional features, such as the presence of intracellular compartments bounded by internal membranes. The Verrucomicrobiota comprises microorganisms from various ecological niches, including sponge symbionts and soil inhabitants (Kamneva et al., 2012). The main taxonomic classes include three formally defined groups: Verrucomicrobiae, Spartobacteria, and Opitutae, in addition to two other putative groups, including formally undefined microorganisms. According to *Bergey’s Manual, Akkermansia* is the only characterized genus of the Akkermansiaceae family, a member of the Verrucomicrobiales order (Hedlund and Derrien, 2015); other genera found in the Verrucomicrobiales are *Verrucomicrobium, Prosthecobacter, Luteolibacter, Roseibacillus, Percisirhabdus*, and *Rubritalea*. All those groups mentioned above comprise free-living bacteria from soil and marine environments, except for *Rubritalea*, a genus comprising sponge symbionts (Hedlund et al., 2015). Both 16S rRNA-based phylogeny, and phylogenomic approaches confirmed the phylogenetic relationship of *Akkermansia* with other members from Verrucomicrobiales (Kamneva et al., 2012).

The phylogenetic context of *Akkermansia* in the Verrucomicrobiota taxonomic tree can be utilized as a case study of how ancient genomic changes sculpted the origin of a gut colonizer. Previous studies have proposed that habitat changes, rather than the phylogenetic background, are preponderant factors defining the pangenome content of a microbial group (Maistrenko et al., 2020). This feature may implicate that *Akkermansia* experienced massive evolutionary changes during its emergence and separation from their non-gut colonizer relatives, including massive gene gain and loss events, or also the emergence of different evolutionary pressures on certain lineage-specific genes. However, it is yet to be established how early or late those events occurred to make *Akkermansia* a professional gut colonizer. The implications of the elucidation of these transformations can be harnessed not only for evolutionary reasons but also for biotechnology implications, such as the discovery of genetic factors that could improve gut colonization or the confirmation of metabolic abilities involved in the proper gut colonization process.

In this study, in order to understand the (pan)genomic transformations involved in the divergence of a gut colonizer from free-living relatives, we performed a phylogenomic analysis of a set of more than three hundred *Akkermansia* genomes and metagenome-assembled genomes (MAGs), calculating the pangenome of different *Akkermansia* genomic species, searching for genes that could be involved in the differentiation of this genus, and calculating the evolutionary pressure of several genes from the most represented genomic species.

## 2. Methodology

### 2.1. Genome dataset definition

A set of six hundred candidate genomes classified the Akkermansiaceae family was downloaded from the NCBI Genbank FTP site (May 2022). Those genomes were evaluated using two criteria: their taxonomic identity and their degree of completeness and contamination. The taxonomic identity was confirmed by using the program ‘*classify_wf*’ of the GTDB-TK program, version 2.1.0 (Chaumeil et al., 2019), using the database release 207 as the reference, selecting all genomes classified into the *Akkermansia* genus (“g__Akkermansia”). Genome completeness and contamination were calculated using the program ‘*lineage_wf*’ from CheckM version 1.1.3 (Parks et al., 2015); only those genomes with completeness equal to or higher than 90%, and contamination below 5%, were selected, according to current recommendations (Bowers et al., 2017).

### 2.2. Annotation, identification of orthogroups, and phylogenomic tree

All members of the final dataset were annotated *de novo* using Prokka, version 1.11 (Seemann, 2014) (relevant parameters: *--metagenome --kingdom Bacteria --addgenes*). Orthogroups from the set of the final dataset were calculated by Orthofinder version 2.5.5 (Emms and Kelly, 2019) with the ‘*-og*’ parameter. In order to make a phylogenetic tree for the *Akkermansia* plus the outgroup genomes, a multiple sequence alignment was constructed from a set of 87 concatenated, single-copy conserved orthogroups by using MAFFT version 7.490 (parameters: *--maxiterate 1000 --localpair*) (Katoh et al., 2019); the alignment was used by *iqtree* version 2.1.4 (Nguyen et al., 2015) (parameters: *-m TEST --alrt 1000*) to generate a maximum likelihood-based tree with an aLRT with 1,000 replicates as the branch support test. The phylogenomic tree was visualized using the *Toytree* Python package (Eaton, 2020).

### 2.3. Definition of genomic species

In order to detect the genomic species represented in the *Akkermansia* selected dataset, we combined the prediction from GTDB-tk (see above) with the prediction of clusters defined by average nucleotide identity (ANI) values. All genomes were compared in an all-vs-all manner using FastANI version 1.32 (Jain et al., 2018) with default parameters. The raw pairwise comparison data was filtered, discarding all ANI values below 95%, the classical intra-species boundary for microbial genomes (Richter and Rosselló-Móra, 2009). Filtered pairwise comparisons were analyzed by the *MCL* program, creating putative genomic species clusters in an analogous manner as observed in network clustering (van Dongen and Abreu-Goodger, 2012).

### 2.4. Pangenome analysis

In order to analyze the pangenome of the main *Akkermansia* genomic species groups, separated Orthofinder executions were performed with the proteomes from each cluster without any outgroup (parameters: *-M msa -y*). The orthogroup matrix (including unassigned orthogroups) was obtained for each run and utilized for different pangenome metrics. Pangenome curves were created using the *panplots* function in R (created by *SioStef*)^1^, using 1,000 permutations. The γ value from the Power law mentioned by Tettelin et al. (2008) was also calculated for each pangenome, using the function *curve_fit* from *scipy* python package, using the equation *“y* = *ax^𝛾^,”* applied on the *panplots* output. We calculated the shell, cloud, “soft-core” and core components of the pangenome from the complete orthogroup matrix by using Python scripts with the *pandas* package, considering the following criteria: *core* gene families as the orthogroups present in 100% of the strains, soft-core groups as present in between 99.999 and 90% of the strains, shell as groups present between 89 and 15% of strains, and cloud as the gene families present in between 14% and the equivalent to two strains. Unique groups can be deduced from the set of “species-specific orthogroups,” and the “unassigned genes,” both reported by Orthofinder. Figures were created with *ggplot2* and the *ggarrangment* packages.

### 2.5. Functional annotation

*Akkermansia* proteomes were analyzed using EggNOG mapper version 2.1.6 (Cantalapiedra et al., 2021) (parameters: “--tax_scope_mode narrowest --tax_scope prokaryota_broad --go_evidence experimental”). COGs at the *root* level were extracted for those classifications, and the categories were established according to the current COG version.^2^ Carbohydrate active enzymes (Flint et al., 2012), including glycoside hydrolases (GH), were searched using the HMM database (v. 11) from dbCAN, the search tool based on CAZy (Zhang et al., 2018), using an e-value <1e-10. Proteases were searched in the dataset using the MEROPS Database Release 12.4 (Rawlings et al., 2018). Sulfatases involved in mucin desulfation were searched by using the SulfAtlas database version 2.3.1 as the reference (Stam et al., 2023). MEROPS and SulfAtlas searches were performed by using Diamond (Buchfink et al., 2015) *blastp* searches using the *Akkermansia* protein dataset as the query and the full-length sequence repository in each case as the database (e-value <1e-10, subject coverage >70%).

### 2.6. Gene gain/loss model for combined pangenomes

In order to create a general gene gain/loss model for *Akkermansia*, the most conserved (core + soft-core) of each species cluster (or the current proteome for 1-genome species groups) were compared by using Orthofinder as previously mentioned. The generated orthogroup matrix was utilized for the generation of a phylogenomic tree, as previously mentioned, confirming that the position of the species in the tree was the same as the one observed in the previous tree. This tree and the binary version of the complete orthogroup matrix were used by the software Count (Csurös, 2010) for the calculation of gene gain/loss rates following the Csûrös - Miklós model, optimized with a Poisson distribution at the root; the rates were also optimized considering a variation across families to 1:1:1:1 gamma categories for the edge length, the loss rate, gain rate, and the duplication rate, respectively. The convergence criteria were set to a likelihood delta of 0.05 with a maximum of 1,000 rounds. The calculated rates were used to generate an analysis following Wagner parsimony using the same penalty score (equal to 1) for gains and losses. The final tree was represented by using the ETE3 python package (Huerta-Cepas et al., 2016). By using ETE3 as well, the tree structure and the Count output were compared in order to identify the orthogroups present and absent in each LCA or leaf (extant genomic species). The orthogroups were also compared to the EGGNOG-mapper output to define the functional content of each LCA.

### 2.7. Evolutionary metrics: Tajima D and dN/dS ratios

In order to calculate Tajima’s D value, the nucleotide sequences from the coding sequences of those proteins found in the core and soft-core genes from the top 4 pangenomes analyzed above were utilized. Sequences were aligned with MAFFT, as mentioned above. Nucleotide multiple alignments were utilized to calculate Tajima’s D values using the *tajima.test* function from *pegas* R package.^3^ Graphs were created with *ggplot2*.

In order to calculate dN/dS ratios, pairwise values were calculated for an aligned set of orthogroups conserved among four genomic species (against the CDS content from *A. glycaniphila* PyT as the outgroup). Alignments were converted into codon-aware aligned using PAL2NAL (Suyama et al., 2006). Calculation of dN, dS, and ω was made by using CODEML program from the PAML package (Yang, 2007), using the following parameters: “*runmode = −2, seqtype = 1, CodonFreq = 3, model = 1, NSsites = 0, icode = 0, fix_kappa = 0, kappa = 1, fix_omega = 0, omega = 0.5*.” Additionally, pairwise Tajima-Nei distances were calculated from the same members of each alignment using the *Bio::Align::DNAStatistics* module from BioPerl (Stajich, 2007). Pairwise comparisons with distances equal to zero, dN/dS > 5, and dS > 10 were discarded from the analysis. Pairwise dN/dS and Tajima-Nei distance values were also filtered, considering only orthogroup comparisons between sequences from different genomic species.

### 2.8. Horizontal gene transfer (HGT) calculation

Genes potentially acquired by horizontal gene transfer (HGT) were inferred using HGTector v2.0b3 (Zhu et al., 2014). Selected coding sequences were analyzed against the default reference database (38,488 genomes, retrieved in September 2022); the database was formatted using DIAMOND (Buchfink et al., 2015), retrieving valid hits using default parameters. Sequence hits from Akkermansiaceae (NCBI TaxID 1647988) were considered as *self*.

### 2.9. Statistical tests

All statistical tests were conducted in R version 4.1.0. Normal distribution was evaluated using the Shapiro–Wilk test (*shapiro.test*). We compared data groups from two conditions with non-normal distribution using the Mann–Whitney *U* test (*wilcox.test*).

## 3. Results

### 3.1. The phylogenetic composition of the filtered *Akkermansia* dataset

The final dataset, obtained from the NCBI Genbank repository and filtered by taxonomy and completeness/contamination standards, corresponds to a collection of 367 *Akkermansia* genomes distributed in 25 genomic species clusters (Supplementary Figure 1 and Supplementary Tables 1, 2). Some interesting genomes, such as *Ca.* Akkermansia intestinigallinarum (GCA_019114365.1), could not be included in the final dataset due to their below-cutoff completeness (84.61%, against the UID2982 marker set). Most species clusters were previously detected in the GTDB-TK reference. The four most prevalent clusters represented *Akkermansia muciniphila*, *Akkermansia* sp004167605, *Akkermansia muciniphila*_B, and *Akkermansia* sp001580195. Each cluster contained 193, 73, 40, and 19 genomes, respectively (Supplementary Table 2). In contrast, 13 genomic species groups are represented by only one genome per cluster.

**Figure 1:**
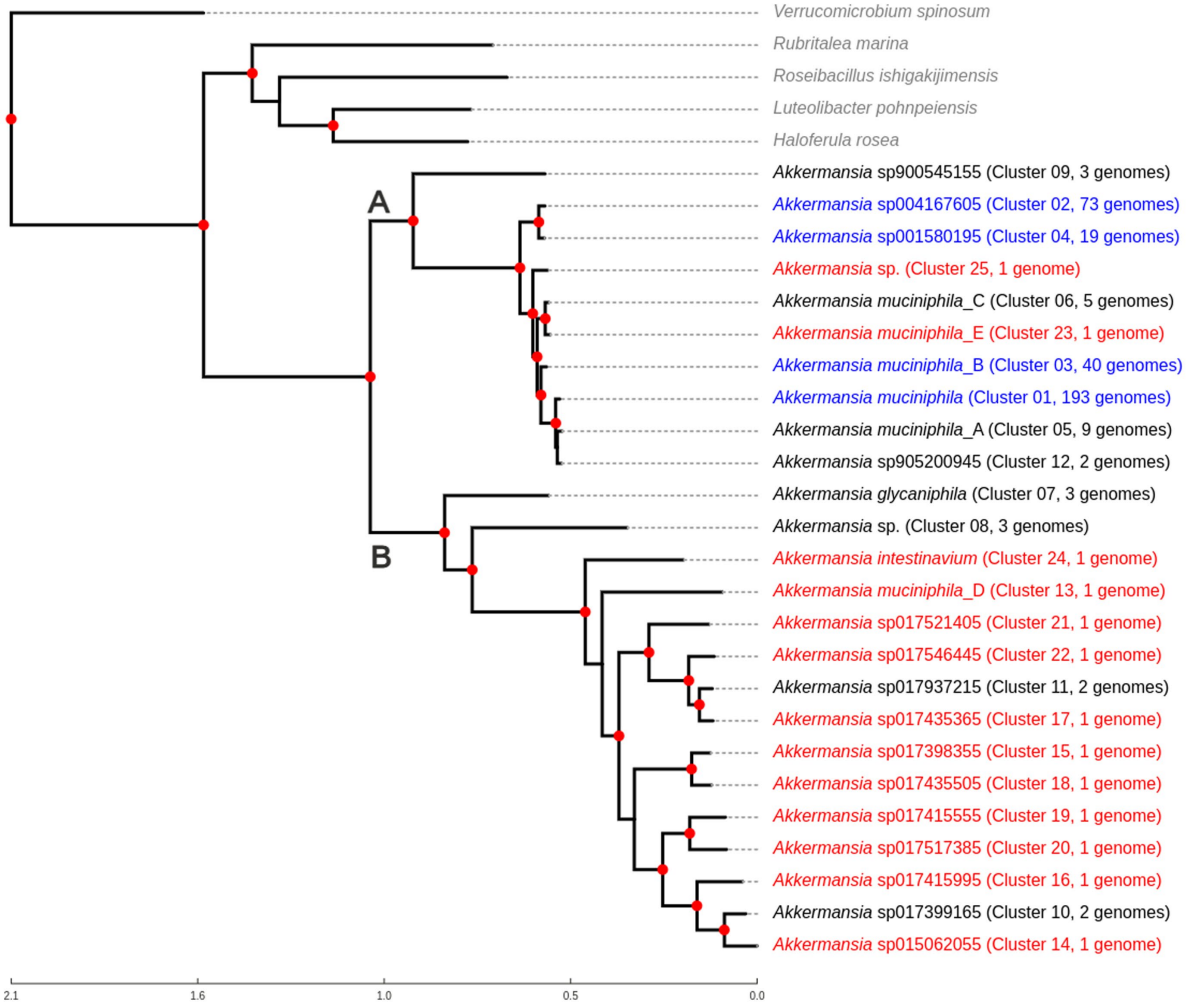
Phylogenomic tree showing the relationship between different *Akkermansia* genomic species, in comparison with other related members of Verrucomicrobiales. The tree was created from the alignment of 87 single-copy conserved protein families, using the maximum likelihood method in IQTREE, with the use of the approximate likelihood ratio test (aLRT) as the branch support test. Red nodes indicate branch support values equal to 100. Taxa in red represent genomic species composed only of one genome; taxa in blue indicate that those genomic species are the top four clusters with the most genomes. Clades “A” and “B” are mentioned in the text.

A representative tree was created from the original dataset (Figure 1), including five outgroups representing different genera of Verrucomicrobiales (also listed in Supplementary Table 1). This rooted tree showed the existence of two main clades (Clades A and B, see Figure 1). The clade A comprised *A. muciniphila* and other related species (GTDB names sp900545155, sp905200945, muciniphila_A, muciniphila_B, muciniphila_E, muciniphila_C, sp001580195, sp004167605, and Cluster25, without no specific species level classification in GTDB). Compared with previous studies (Karcher et al., 2021; Lv et al., 2022), the GTDB *A. muciniphila* genomic species corresponds to the *Amuc I* phylogroup, whereas *Akkermansia muciniphila_B* corresponds to *Amuc II*, *Akkermansia muciniphila_C* to *Amuc III*, and *Akkermansia* sp001580195 to *Amuc IV*. On the other hand, Clade B includes *A. glycaniphila, Ca*. Akkermansia intestinavium, *Akkermansia muciniphila_D*, and several other species, including an undetected cluster in GTDB, called Cluster08. Genetic distances between members of Clade A are significantly lower than the observed distances between members of Clade B (non-normal distribution; *p* < 0.05 according to Mann–Whitney *U* test, data not shown). Unless specified, we used this 367-genome dataset (and its corresponding genome species clusters) for all downstream analyses.

### 3.2. Pangenome properties of the *Akkermansia muciniphila*, sp004167605, *muciniphila*_B, and sp001580195 species clusters

Recently, comparative genomics and *A. muciniphila* pangenome studies have been performed multiple times (van Passel et al., 2011; Guo et al., 2017; Xing et al., 2019; Lv et al., 2020; Geerlings et al., 2021; Karcher et al., 2021; Kim et al., 2022; Li et al., 2022; Lv et al., 2022; Ouwerkerk et al., 2022). However, the use of an Orthofinder-based strategy is not common. In order to assess our strategy in the *Akkermansia* dataset, we performed a pangenome analysis using the four genomic species clusters with the highest number of genomes. For those genomic species, the core-, soft-core, shell, cloud, and unique components and the accumulation curves were calculated from the Orthofinder analysis results, and the γ value from the Power law was also calculated for each pangenome (see above).

**Figure 2:**
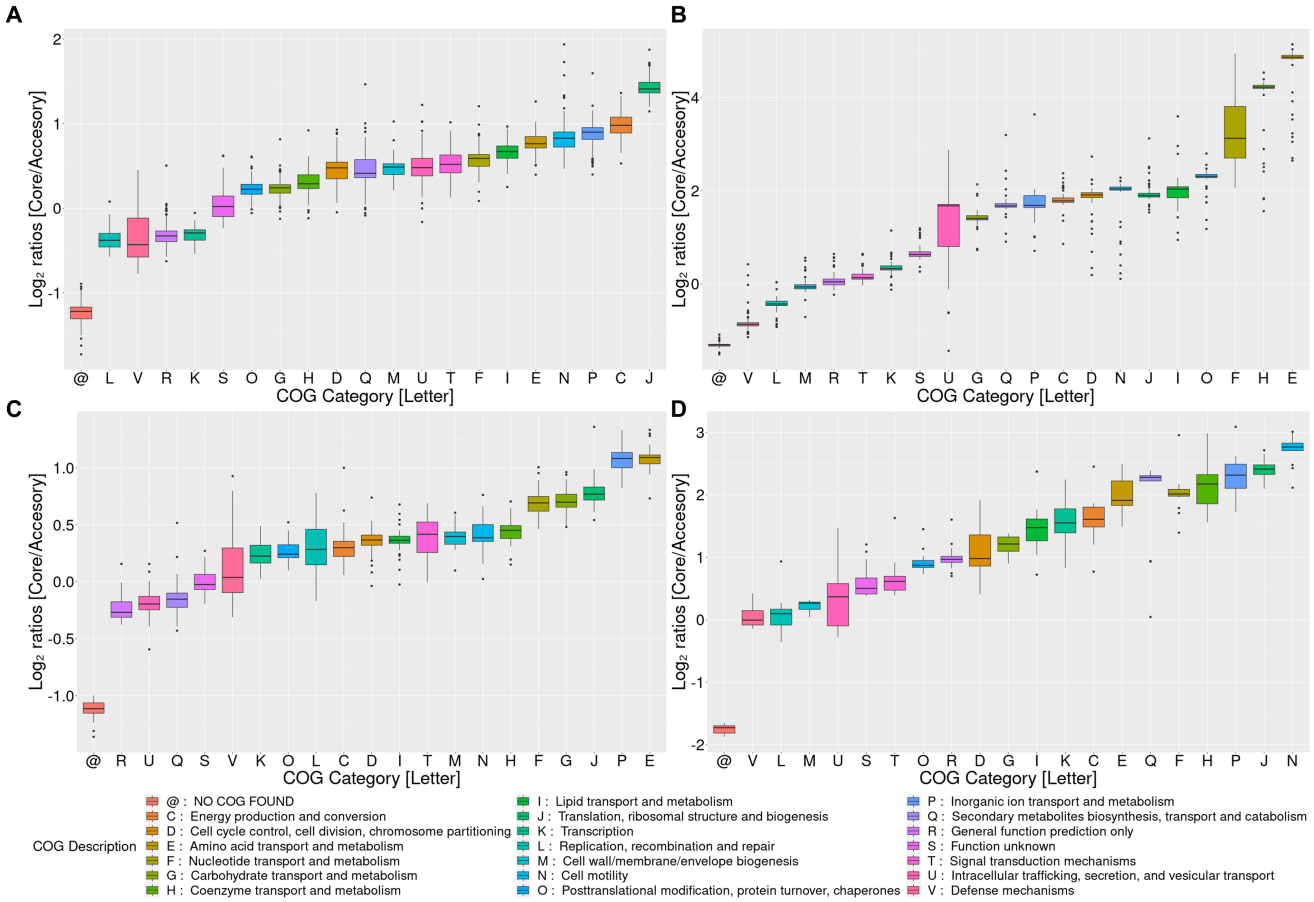
Pangenome analysis for the top four *Akkermansia* genomic species clusters with the most members. Log2 ratios of the percentage of core versus accessory (the sum of soft-core, shell, cloud, and uniques) genes for each COG category among pangenomes. Clusters I, II, III, and IV are represented by panels **(A–D)**, respectively. COG category descriptions are represented in the lower panel @: gene families without any COG.

This analysis showed that the core genome for those genomes ranged between 2,170 and 1,265 orthogroups (Figure 2 and Table 1). The core genome set from Cluster 2 (*Akkermansia* sp004167605) is the longest among the analyzed species, and Cluster 1 (*A. muciniphila*) contained the smallest core genome. The pangenome comprised between 6,865 and 3,515 orthogroups among those four species; these changes in pangenome content seem to depend on the number of genomes in each cluster, a feature seen in other open pangenome models (Costa et al., 2020). The curve profile of the pangenome accumulation plot (Supplementary Figures 2A–D and Table 1) exhibited the properties of an open pangenome (0 < γ < 1) for all four genomic species, confirming this feature observed in other studies, observing the pangenomes for the Amuc I to Amuc IV phylotypes (Bukhari et al., 2022).

**Table 1:** Main statistics from pangenome analysis for the top four genomic clusters found in the *Akkermansia* dataset.

	Cluster 1 (*A. muciniphila*)	Cluster 2 (A.sp004167605)	Cluster 3 (*A. muciniphila*_B)	Cluster 4 (A.sp001580195)
Total genomes	193	73	40	19
Total pangenome genes	6,865	4,622	4,341	3,515
Core genes (in 100% of genomes)	1,265	2,170	1,462	1,936
Soft-core genes (less than 100%, more than 90%)	666	370	733	249
Shell genes (less than 90%, more than 15%)	642	110	806	769
Cloud genes (less than 15%, at least in two genomes)	1,905	589	551	154
Unique genes (in just one genome)	2,387	1,383	789	407
Gamma value (Power law)	0.2637	0.18199	0.15516	0.10665

The openness of those four pangenomes requires addressing whether the core/soft-core contents are functionally different compared to the shell, cloud, or unique genes. To observe these differences, we compared the percentage of genes assigned to each COG category between the core genome pangenome and the core, soft-core, cloud, shell, or unique fractions (Supplementary Figures 3A–D), as well to obtain the Log2 ratios between the core and the “accessory” (the sum of soft-core, shell, cloud, and unique) pangenome fractions (Figures 2A–D). Functional categories where the percentage of genes assigned in the core pangenome is two-fold (log2 equal or higher than 1) were considered core-enriched categories. On the other hand, functional COG categories where the percentage of genes assigned to the core pangenome was one-half (log2 equal or lower than −1) of the accessory fraction were considered core-depleted.

These analyses showed some general tendencies; for example, categories associated with metabolism (e.g., categories C, G, E, F, H) and information transfer (e.g., J, L, K) were the most prevalent in the core and soft-core fractions. On the other hand, categories such as X (“Mobilome”) or Q (“Secondary metabolite transport and metabolism”) had the highest percentages detected in the shell cloud or unique gene fractions. Genes without COGs have strongly higher percentages in the fraction of unique genes (data not shown). Cluster 2 and Cluster 4 were the genomic species with the highest number of core-enriched categories compared to the accessory genes. COG categories U, G, Q, P, C, D, N, F, H, and E showed high core-to-accessory ratios in Cluster 2, while Cluster 4 showed I, K, C, E, Q, F, H, P, J, and N presenting the largest ratios. The categories that overlap these two Clusters correspond to P, C, F, H, and N. Cluster 1 showed that category J was core-enriched, and Cluster 3 showed that P and E categories were core-enriched (Figures 2A–D). Category N (“Cell motility”) is highly core-enriched in both Cluster 2 and Cluster 4. Motility-associated genes in those species were related to pili synthesis as an outer-membrane protein, suggesting that these clusters could differentiate from the others due to their differences in host-microorganism membrane interactions (Ottman et al., 2016), which can be specific depending on the host organism. Furthermore, category C was also enriched in the core group of genes in Clusters 2 and 4, suggesting that “Energy production and conversion” might be one of the key functions in the genomic differentiation of *Akkermansia* species. Since all those four Clusters are strongly related, and Clusters 2 and 4 shared a direct common ancestor (belonging to Lineage A, see Figure 1), those differences could respond to evolutionary differences between those organisms and the other two analyzed clusters. This final observation also suggests the need to analyze the evolutionary changes of the gene content between different organisms across *Akkermansia* evolution.

### 3.3. Gene gain/loss model analysis of *Akkermansia* lineages and the reconstruction of the gene composition and functionality of *LAkkCA* (the Last *Akkermansia* Common Ancestor)

**Figure 3:**
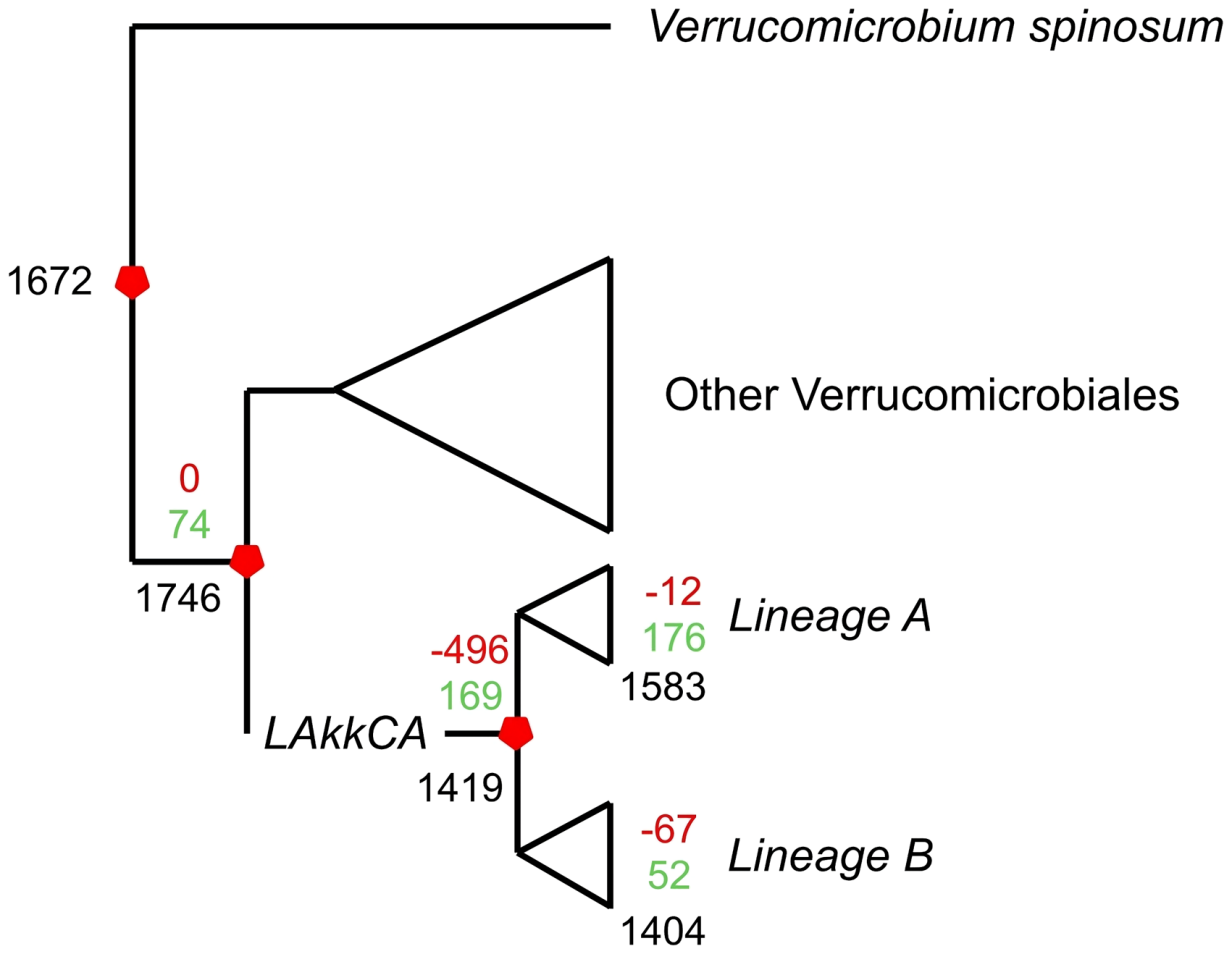
Overview of the gene gain/loss model representing gene content changes across the evolution of the *Akkermansia* genus. The phylogenomic tree from Figure 1 was combined with the orthogroup matrix generated from the Orthofinder output, and analyzed by Count. Black numbers represent the number of shared families for a given ancestor or extant genome; green and red numbers represent gained or missed gene families, respectively.

As seen previously, the 367 *Akkermansia* genome dataset utilized in this study represented 25 potential genomic species (Figure 1), as some of their pangenome properties could be related to evolutionary patterns (see above). To study the evolutive gene gain/loss dynamics across the evolution of this genus, we predict the content of the different common ancestors, from the most recent common ancestors to the putative “Last *Akkermansia* Common Ancestor” (from now, *LAkkCA*), in comparison with a set of members of *Rubritalea*, *Roseibacillus*, *Luteolibacter* and *Haloferula*, the closest Verrucomicrobiales as outgroups (Figures 1, 3 and Supplementary Figure 4). This comparison was performed with core and soft-core gene sets within species groups since this comparison focused on ancestral changes rather than more recent changes.

According to the prediction, *LAkkCA* contained 1,419 genes, including 169 gained genes compared to its non-*Akkermansia* ancestor (Figure 3). The prediction also showed that the last common ancestors (LCAs) from Lineages A and B (Figure 1) experienced subsequent gene gain/loss events: 176 gained/12 missed genes in Lineage-A LCA, and 52 gained/67 missed genes in Lineage-B LCA. The median of gene loss events in Lineage A was lower than the median in Lineage B (*p* < 0.05 according to Mann–Whitney *U* test, data not shown). This feature suggests that Lineage A was more conservative than Lineage B in its gene content. In general, gene acquirements were most frequent than gene loss events across the *Akkermansia* phylogeny.

**Figure 4:**
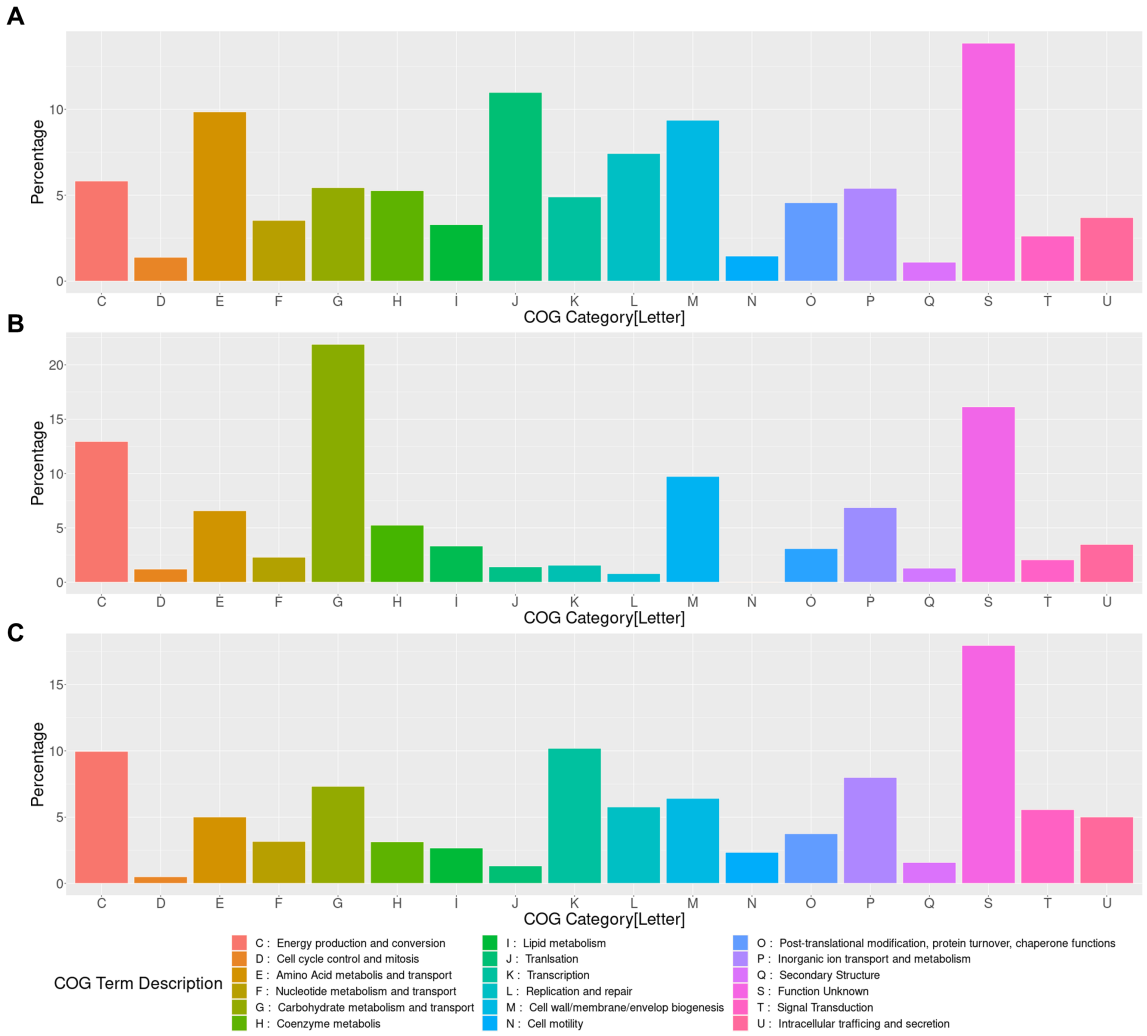
Functional characterization of the genes content present, gained and lost in *LAkkCA*. **(A)** Percentage of assigned genes to each COG category in the predicted total gene content of *LAkkCA*. **(B)** Percentage of assigned genes to each COG category in the sub-dataset of gained genes in *LAkkCA* (genes present in this LCA but absent from its ancestral node). **(C)** Percentage of assigned genes to each COG category in the sub-dataset of those genes absent in *LAkkCA*, in comparison with their ancestor node. COG category descriptions are represented in the lower panel.

The functional profile of those genes showed that the gene content of *LAkkCA* (Figure 4A), expectedly, included an important percentage of genes in several essential processes such as Translation (category J), DNA Replication (category L), and Transcription (category K), as a signal of the role of those essential processes in cellular configuration. Genes involved in metabolism (COG categories C, E, F, G, H, I) were found in important proportions. Notoriously, genes involved in Carbohydrate Metabolism (Category G, Figure 4B) corresponded to a significant fraction of genes acquired in *LAkkCA*; in counterpart, genes from the J, K, and L categories were detected in lower fractions among the acquired genes (Figure 4B). This latter observation concurs with previous analyses in different models showing that those kinds of functions were most reluctant to be transferred horizontally, at least between distant groups (Kanhere and Vingron, 2009). Additionally, a gene set of 142 genes, without any COG assignment, was detected in *LAkkCA*.

In addition to the gene set acquired by *LAkkCA*, there is a set of genes that *LAkkCA* lost during its differentiation from their non-Akkermansia relatives (Figure 4C). According to the gene gain/loss model, 496 genes were lost by the ancestor of the *Akkermansia* genus (Figure 3 and Supplementary Figure 4). These changes could be associated with adaptions to the new niche; for example, whereas nearly %1 of the genes acquired by *LAkkCA* belonged to the “Transcription” (K) category (Figure 4B), almost 10% of the missed genes by *LAkkCA* belonged to this same category (Figure 4C), suggesting that the change of niche could involve changes in the transcriptional regulation program of the emergent genus.

### 3.4. Predicted metabolism and genetic features of *LAkkCA*

The prediction of the putative genetic content that harbored the hypothetical last common ancestor of this complete genus raises the opportunity to predict the properties of early members of the *Akkermansia* genus. We annotate the putative gene content from *LAkkCA* with current state-of-the-art tools to proceed with this prediction. The predicted functional repertoire of *LAkkCA* (Supplementary Table 3) includes the capability to encode genes for the NADH:quinone dehydrogenase, the Cytochrome bd-type quinol oxidase [a terminal oxidase with high oxygen affinity (Borisov et al., 2021)], and the ATPase complexes, in addition to a relatively complete central carbon metabolism (including glycolysis, a partial TCA cycle, and the reductive branch of the pentose phosphate pathway). The genetic capabilities of *LAkkCA* also include the biosynthesis of some phospholipids, such as phosphatidyl glycerol, phosphatidylserine, phosphatidylethanolamine, and cardiolipin. The prediction of *LAkkCA* metabolism also suggests the production of acetate via pyruvate dehydrogenase [EC:1.2.5.1] (K00156) and the production of propionyl-CoA via propionyl CoA:succinate CoA transferase (COG0427). The possession of NADH dehydrogenase, ATPase, and some terminal oxidases suggest an inheritance of the respiratory metabolism from their non-*Akkermansia* ancestors, something noticeable if we consider that previous studies suggested that *A. muciniphila* may conduct microaerobic metabolism in the mucus layer niche (Ouwerkerk et al., 2016b), even if is also capable of performing anaerobic metabolism. In concordance with this ability to use oxygen, *LAkkCA* also contained several genes involved in oxidative stress: several genes encoding members of COG0526 (Thiol-disulfide isomerase or thioredoxin), in addition to a member of COG1225 (Bcp Peroxiredoxin), COG0450 (AhpC peroxiredoxin), COG1592 (Rubreythrin), COG0605 (Superoxide dismutase) and COG0753 (KatE Catalase). This latter finding suggests the need for a proper adaptation to higher oxygen levels during the origin of the *Akkermansia* genus.

According to the prediction of its genetic content, *LAkkCA* also contained a series of proteins involved in its relationship with the host and environment. For example, this ancestor contained a set of genes encoding proteins containing COG0666 (“Ankyrin repeat,” ANKR), a ubiquitous domain involved in protein–protein interactions. In Bacteria, ANKRs were mostly investigated in proteobacterial organisms, especially pathogens, where those proteins could be associated with protein secretion systems involved in pathogenic interactions with the host (Al-Khodor et al., 2010). In the case of *Akkermansia*, those proteins could be part of a set of secreted proteins that could generate an effect on the host, as seen with other secreted and exposed proteins detected in *A. muciniphila* (Vidal-Veuthey et al., 2022). *LAkkCA* also encoded a predicted set of genes for Type IV pilus assembly, suggesting a role of this ubiquitous complex, involved in several functions such as motility, biofilm formation, and adherence (Ligthart et al., 2020), in the early adaptation of this genus to the gut environment. Moreover, *LAkkCA* also encodes a couple of genes encoding putative autotransporters; those proteins contain their own exportation system, transporting one domain (the “passenger” domain) of the same protein across the outer membrane of Gram-negative bacteria (Clarke et al., 2022). Our prediction suggests that those two autotransporters may be involved in mucin degradation and adherence, since their passenger domains were a sialidase (GH33) domain and an adhesin, respectively (data not shown).

### 3.5. Insights into the mucin metabolism in *LAkkCA* and its evolution across the *Akkermansia* genus

**Figure 5:**
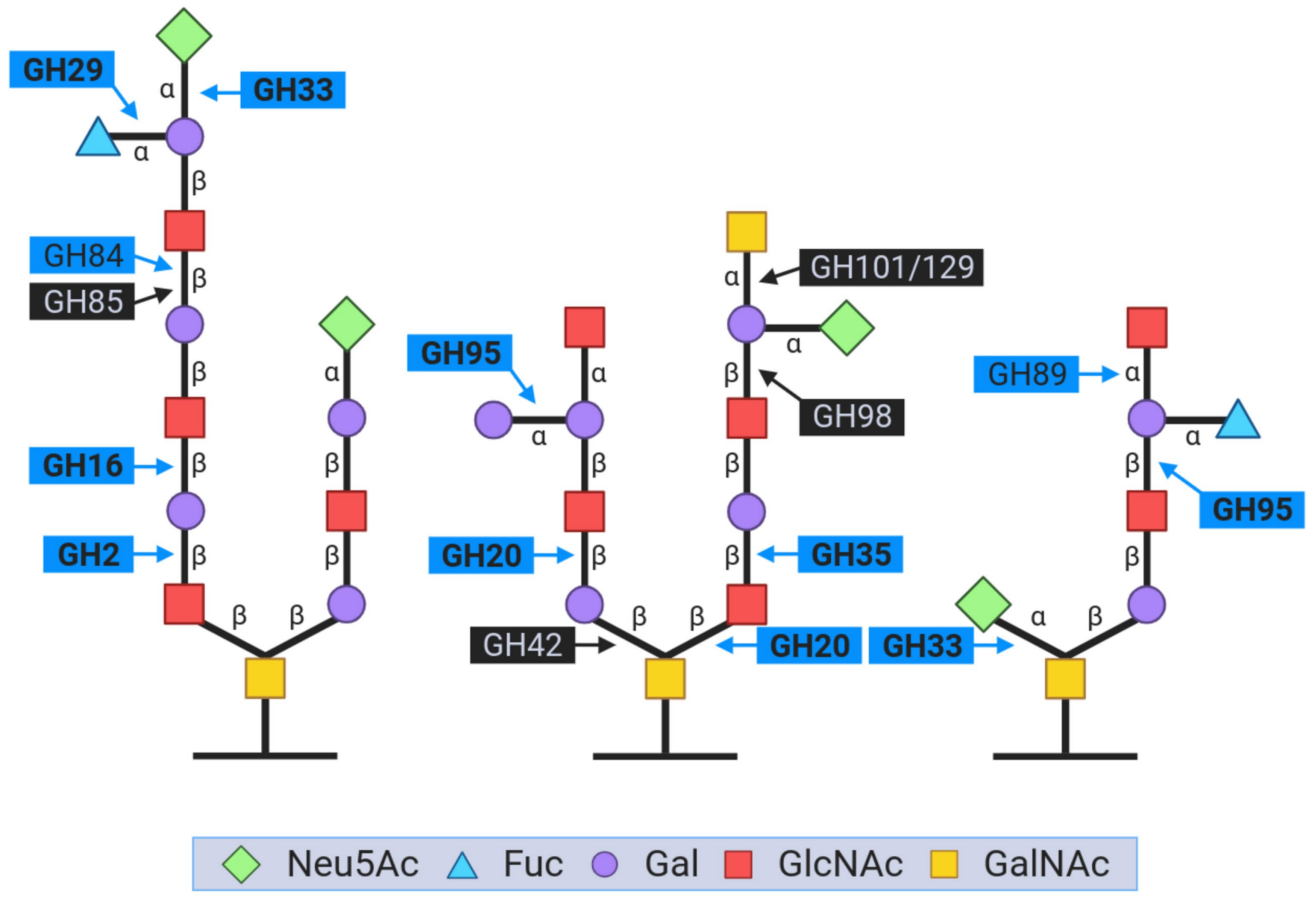
Representation of the key glycoside hydrolase (GH) families involved in the breakdown of carbohydrate moieties during mucin degradation. The figure is inspired by the content from Glover et al. (2022). The GH names in bold are those that were found in *LAkkCA*. GH names closed by blue boxes corresponded to the enzymes detected in *Akkermansia* genomes. GH names closed by black boxes corresponded to the enzymes absent in *LAkkCA* and *Akkermansia* genomes.

As mentioned earlier, mucin metabolism is a distinctive feature of *Akkermansia* species, and some studies have suggested that *A. muciniphila* showed higher growth in mucin than in glucose (Derrien et al., 2004; Glover et al., 2022). A set of carbohydrate-active enzymes (CAZymes) are commonly associated with glycan moieties degradation in mucin. Those CAZymes are important players in the gut microbiome, and several key GHs are identified in *Akkermansia* involved in its ability to degrade mucin (Chen et al., 2019; Glover et al., 2022). We searched for enzymes involved in the degradation of this glycoprotein in *LAkkCA*, the predicted ancestors for Lineages A and B, and the representative content for each species cluster (Supplementary Table 4). We found that genes encoding some CAZyme families involved in mucin degradation, such as GH20 (related to β-hexosaminidases and β-1,6-N-acetylglucosaminidases), GH29 (related to α-fucosidases), GH33 (related to neuraminidase and sialidases), GH35 (β-galactosidases/β-glucosaminidases), and GH95 (also related to α-L-fucosidases), were detected in *LAkkCA* (Figure 5). Moreover, those same functions were remarkably absent from the non-*Akkermansia* relatives (data not shown). In this ancestor, we also found another CAZyme family called GH16_3. This GH family comprises O-glycanase capable of targeting the polyLacNAc structures (consists of repeated N-acetyllactosamine units) within oligosaccharide side chains of both animal and human mucins (Crouch et al., 2020). Most GHs found in *LAkkCA* were also found most, if not all, *Akkermansia* species, experienced in some cases the inclusion of multiple copies, such as the case of GH20 (Supplementary Table 4). These analyses strongly suggest that the origin of *Akkermansia* involved the possession of a basic set of glycan mucin degradation, and that gene set was strongly conserved or even amplified.

Subsequently, despite being absent in *LAkkCA*, other relevant CAZymes such as GH2 (mainly composed by beta-galactosidases), GH84 (N-acetyl β-glucosaminidase), GH89 (α-N- acetylglucosaminidase), and GH110 (α-galactosidase), were also found in *Akkermansia* species as expected (Glover et al., 2022). For example, a copy for GH2 was predicted in Lineage-A LCA and among different *Akkermansia* species, GH2 was also detected, often in multiple copies (Supplementary Table 4). The amplification of the number of copies of certain GHs involved in mucin may reflect the reinforcing role of these enzymes in this important feature. Moreover, it may also reflect clade-specific or lineage-specific adaptations from different *Akkermansia* species to adapt to the mucin complexity found in different animals, from reptiles to mammals (Belzer and de Vos, 2012; Ottman et al., 2017). The research of phylogenetic patterns in those GH families may gain some insights into the processes of functional diversification that *LAkkCA* suffered until the current day representatives.

CAZymes are not the only relevant component to search in the mucin degradation machinery. Since mucin is a glycoprotein, the peptidase repertoire is also important to explore among *Akkermansia* genomes. Additionally, the ability of the gut microbiome to degrade peptides is a well-reported phenomenon (Wallace and McKain, 1997). Protease activities in the human gut are relevant for several microbial community members since they could be involved in the use of nutrients, host interactions, and even connections with host health and disease (Caminero et al., 2023). To execute this search, the same representative genetic content from the 25 genomic species, as well as the content of *LAkkCA* and the Lineages A- and B- LCAs, were compared against MEROPS (Rawlings et al., 2018). Since mucin is commonly sulfated, we also searched for sulfatases by using SulfAtlas as a reference (Stam et al., 2023). The search for peptidases and sulfatases associated with mucin metabolism in *Akkermansia* species showed that several of those enzymes were conserved among the genus and found in *LAkkCA* (Supplementary Table 5). For example, peptidases from the metallopeptidase family (M03A, M15D, M20F, M24A, M24B, M38, M42, M50B), serine proteases (S26A, S33, S41A, S54), cysteine proteases (C26, C82A) and other types (U32, I04, A24A, T05), and were detected in *LAkkCA*. Some of them, such as S26A (signal peptidase I), A24A (type 4 prepilin peptidase), and S41A (C-terminal processing peptidase-1), for example, seem to have housekeeping functions. In contrast, some families such as U32 (collagenase), I04 (alpha-1-peptidase inhibitor), or M42 (related to tetrahedral aminopeptidases), seem to be acquired as special adaptations. For example, bacterial collagenases have been associated with pathogenic bacteria (Duarte et al., 2016), but no information is available about the role of this peptidase in gut commensals. However, the U32 family was also detected in members of *Prevotella* and *Paraprevotella*, common gut inhabitants, suggesting a common role in the gut environment (Patra and Yu, 2022). Notably, peptidases from the M03A family were found not only in *LAkkCA* and in Lineages A and B LCAs, but also present in multiple copies in all the 25 species, in an analogous manner like GH20. This feature may suggest that this particular peptidase family could have a role in the ability of *Akkermansia* species to colonize the gut, or also that these peptidases could be involved in mucin degradation. The functional importance of these proteases in *Akkermansia* physiology could be related to their ability to interact with host cells, although their exact role remains to be established. The search for sulfatases showed that all members, including *LAkkCA*, Lineages A and B LCAs and the core/representative genome from all *Akkermansia* species, contained a uniform, well-conserved sulfatase patrimony (Supplementary Table 5), including members of sulfatase subfamilies S1-4 (containing [Colonic mucin]-endo-D-Galactose-3-sulfate 3-O-sulfohydrolases), S1-11 (including [mucin]-exo-N-acetyl-D-glucosamine-6-sulfate 6-O-sulfohydrolases), S1-15 (including [mucin]-D-Galactose-6-sulfate/N-acetyl-D-galactosamine-6-sulfate 6-O-sulfohydrolase), S1-16 (mucin- D-Galactose-4-sulfate/N-acetyl-D-galactosamine-4-sulfate 4-O-sulfo hydrolase) and S1-20 (inclding mucin- D-Galactose-3-sulfate 3-O-sulfohydrolases and N-acetyl-D-galactosamine-3-sulfate 3-O-sulfatases). This finding has a strong coincidence with the case of the CAZymes: peptidases putatively involved in mucin metabolism have strong conservation among the genus and even are predicted to be present in the last common ancestor of the *Akkermansia* genus.

### 3.6. Prediction of horizontal gene transfer (HGT) events in the different members of the *Akkermansia* genus

The acquisition of new genes can be explained by three main evolutionary processes, *de novo* gene gain, gene duplication, and HGT (Douglas and Langille, 2019). The birth of *de novo* genes can occur at any time in the evolutionary history of a microorganism, detecting these events in ancient genes is complex since it has been shown that rapidly evolving orthologous genes are often not detected in distant taxa, and they tend to be misclassified as *de novo* genes (Elhaik et al., 2006; Van Oss and Carvunis, 2019). Likewise, it has been shown that the adaptation of bacteria to new environments is mainly mediated by the expansion of protein families encoded by genes obtained through HGT and not by gene duplication (Treangen and Rocha, 2011). In this regard, HGT is a source of phenotypic innovation and an important niche adaptation mechanism (Wolska, 2003; Ravenhall et al., 2015). Genetic conjugation, transduction, and transformation are key processes within HGT (Ravenhall et al., 2015), through which genetic material is exchanged between microorganisms that share the same microenvironment despite not sharing a vertical ancestry (Soucy et al., 2015). Therefore, HGT provides a potential adaptive advantage in the bacterium accepting the genetic material since it allows rapid gene transfer between distantly related species (Douglas and Langille, 2019), and it seems to be a reasonable alternative to investigate genetic traits acquired by a bacterial population. In this context, we performed an analysis using the HGTector tool, which detects possible genes derived from HGT events to identify the probable taxonomic origin of genes with a considerable signal for HGT. In this case, the Akkermansiaceae family level was considered as the “self group” to detect donors only from clearly distinctive taxonomic groups.

**Figure 6:**
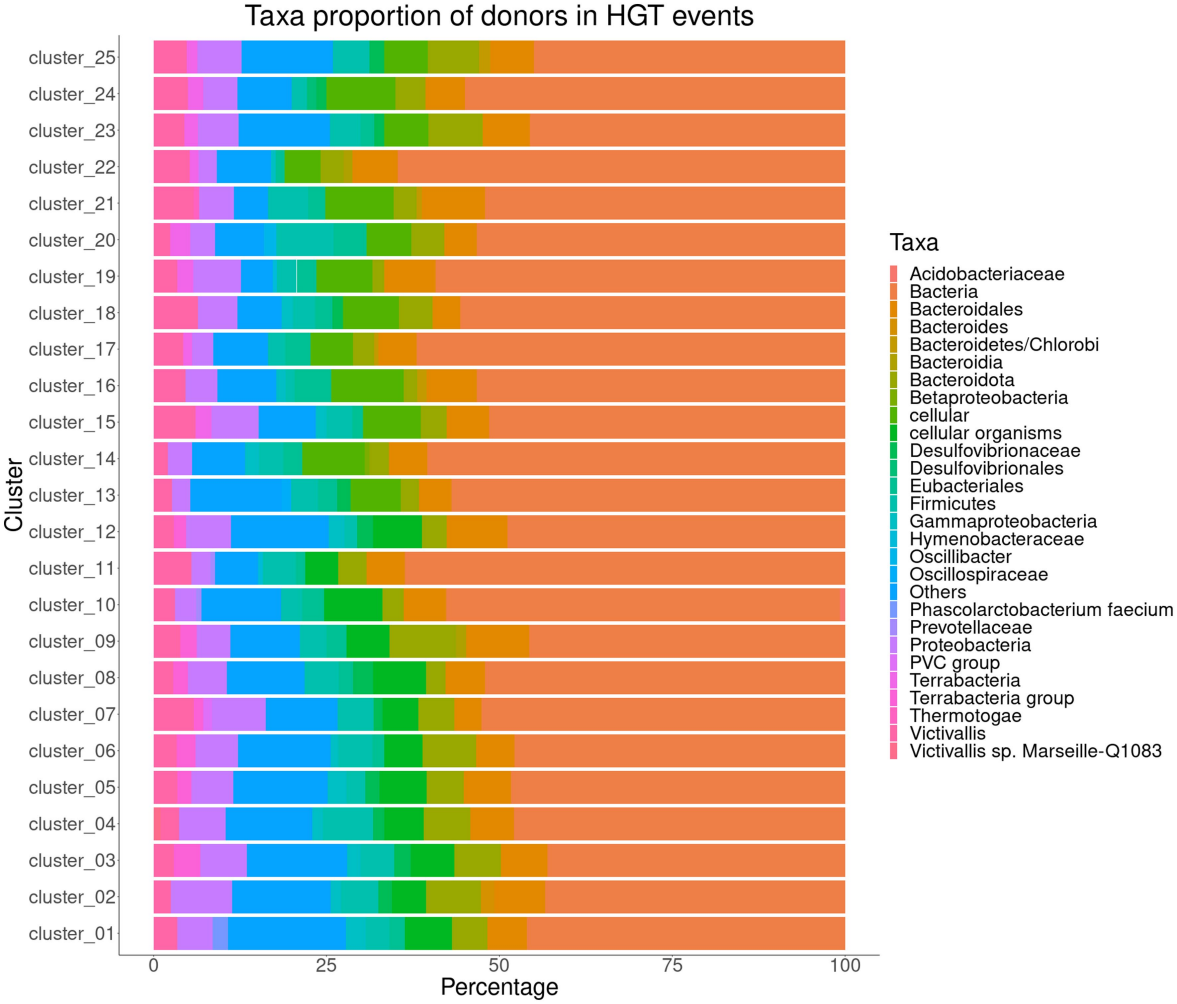
Taxa proportion of donors in Horizontal Gene Transfer events predicted among the representative sequences from 25 clusters (involving the core/soft-core from clusters #1 to #12 and the genomes of the only representative from clusters #13 to #25) representing the genomic species found in the *Akkermansia* dataset.

We predicted genes potentially from HGT for the 25 clusters representing the 367 annotated genomes of *Akkermansia* species. The analysis showed that nearly 6.9 to 10.3% of the predicted genes in the core/soft-core of the cluster 1 to 12 were predicted with an HGT signal, whereas the percentage of genes putatively received by HGT in clusters 13 to 25 ranged between 6.63 to 8.76%. In all genomic species, putative horizontally transferred genes exhibited similar behavior in their putative donors (Figure 6). For example, in all groups, more than 50% of the genes were predicted to have an unresolvable donor from the *Bacteria* domain or from a “cellular organism” (NCBI TaxID 1), followed by members from the *Proteobacteria* and *Bacteroidota* phyla. Interestingly, we detected in the clusters of *Akkermansia* spp. different genes associated with families of glycosyl hydrolase enzymes (GH16, GH31, GH35, and GH57), with a predicted donor from *Bacteria* (superkingdom) and *Bacteroidota*. Some of these enzymes were found in intestinal bacteria such as *Bacteroides plebeius*, *Bifidobacterium longum* subsp. *infantis* and *Fibrobacter* spp. (Qi et al., 2005; Hehemann et al., 2010; Tarracchini et al., 2021), which accounts for possible HGT events. Likewise, in several clusters, detected genes linked to an efflux pump (AcrAB) were also identified with a potential donor from *Proteobacteria*. This efflux pump confers resistance against a wide variety of antimicrobial compounds, such as bile salts, by expelling them out of the cell (Sun et al., 2014). Additionally, AcrAB has been reported in *Escherichia coli* of intestinal origin (Ma et al., 1995), which indicates the probable HGT between different bacterial genera. Consequently, HGT is a process of adaptation to a habitat shared by taxonomically diverse bacterial populations (Zaneveld, 2011; Chen et al., 2021).

HGTector does not determine a direction for gene acquisition from HGT. This is due to the limitations of the reference databases used (Chen et al., 2021), as well as the lack of a systematic phylogenetic analysis, which would help to strengthen the determination of the potential taxonomic origin of the genes acquired by HGT (Zhu et al., 2014). Another drawback is that this type of analysis does not include information regarding the synteny of groups of genes, which, being present in different bacterial taxa may reflect the action of HGT (Lawrence and Roth, 1996; Imam et al., 2011), information on transposable genetic elements is not included either, which is important, since some transposons are shared by Gram-negative and positive bacteria, and could shed light on a possible diversification in the acquisition of genes through HGT (Ojo et al., 2004). In this scenario, it is difficult to trace the probable taxonomic origin of possible HGT-derived genes. Therefore, some questions remain open, such as whether it is possible that *Akkermansia* species have obtained a repertoire of genes through HGT from members of the intestinal microbiota or if these genes originated in the genus *Akkermansia* and were transferred to other intestinal microorganisms, or also if the genes transferred to *Akkermansia* spp. they underwent a specialization process and were later transferred to other taxa. Verifying these situations is an intriguing issue and still to be elucidated.

### 3.7. Tajima distance analyses across the main *Akkermansia* species suggest differential genetic diversity

**Figure 7:**
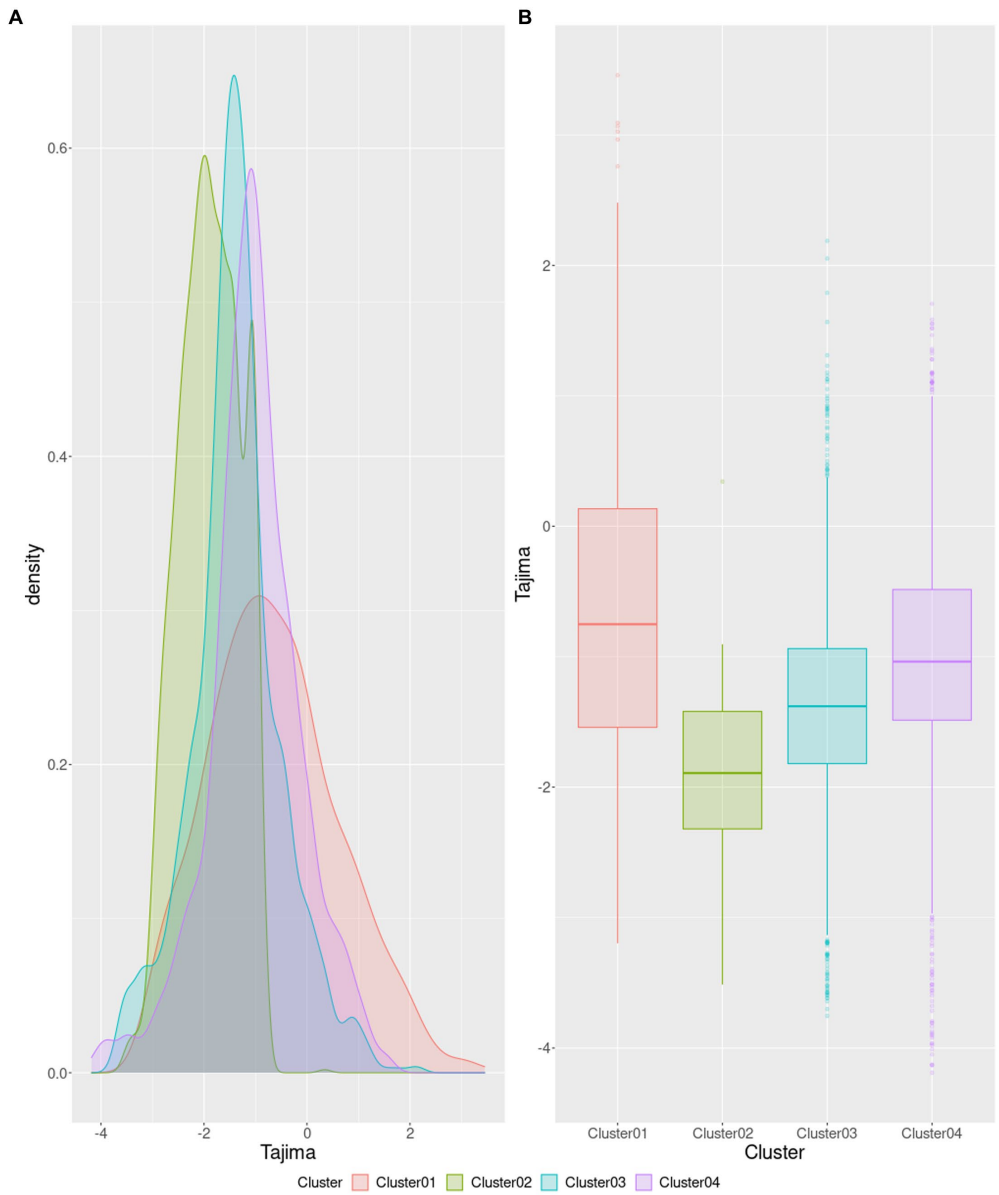
Tajima D distribution among the core gene content among different *Akkermansia* genomic species, presented as density curves **(A)** and boxplots **(B)**.

Tajima D statistical test is a population genetic test used to elucidate if a gene family evolved in a neutral manner, or if it evolved under a non-random process, such as balancing selection, demographic expansion, or contraction, among other effects (Tajima, 1989). In order to evaluate the main tendencies in genetic diversity among the four top *Akkermansia* species clusters (see section 3.2), we calculated the Tajima D values for the core gene families for each species. Our analysis (Figure 7) showed that the distribution of Tajima values had different distributions among the four species (Figure 7A), which Cluster 2 (*Akkermansia* sp004167605) showed a shift to more negative values. Cluster 1 (corresponding to the bonafide *A. muciniphila*) showed a more extended distribution, as seen as well in the boxplots (Figure 7B). In addition, core gene families from Clusters 3 and 4 showed a shift to more positive values in comparison with Cluster 2. The order of median D values was: Cluster1 > Cluster4 > Cluster3 > Cluster2. Using the Mann–Whitney *U* test to make comparisons among two groups, we can found that Clusters #1 and #2 exhibited significant statistical differences between Tajima D values (*p* < 2.07×10^−156^), with a common language effect size (CLES) value of 0.809, which means that 80.9% probability that randomly chosen observation from one group will be greater than a randomly selected from the other group, making this difference substantial. In the same way, in comparison between cluster #1 and cluster #3 and between cluster #1 and cluster #4, both comparisons showed significant differences (*p*-values: 9.59×10^−47^ and 6.01×10^−12^, respectively). However, CLES values show that both comparisons suggest that differences are more modest (CLES of 0.674 and 0.578, respectively). Based on the aforementioned information, our results show that the differences between clusters are sufficient to back up the potential description of new *Akkermansia* species. Differences between cluster #2 versus cluster #3, and cluster #2 versus cluster #4 were also significantly different, although with lower CLES values (*p*-values: 1.35×10^−81^ and 8.12×10^−246^; CLES: 0.30 and 0.18, respectively). This information suggests that the more negative distribution in Cluster 2 is significant in relation to the other three clusters.

The observed more negative distribution in the core set from members of cluster 2 may reflect the properties of their genetic diversity. Highly negative Tajima values (below −2, with a significant *p*-value) may reflect the effect of positive selection since it indicates an excess of rare alleles, suggesting population expansion. In counterpart, very positive D values (above 2) suggest a high proportion of common alleles, indicating balancing selection or population contraction (Carlson et al., 2005). In the case of Cluster #1, the high amplitude of the distribution could suggest a more neutral evolutionary pattern among gene families, although this may also be affected by sample size (Cluster #1 have the most genomes). Therefore, the available information on those four *Akkermansia* genomic species suggests that each one exhibits different selection pressures, as a reflection of their roles in the GI tract.

Since these gene families (orthogroups) were annotated with EggNOG mapper, a posterior approach was the classification of Tajima value distribution across different COGs categories based on each cluster (Supplementary Figure 5). It is possible to observe that for Clusters #1 and #4, a great proportion of categories showed a distribution toward moderate values (closer to zero), suggesting that DNA-sequence changes were nearly neutral in those species clusters (Carlson et al., 2005). However, in Clusters #2 and #3, this analysis showed that genes related to the defense mechanism in bacteria or cell motility (V and N categories, respectively) were not following the neutral theory of molecular evolution. These two categories are commonly found to unfollow the neutral theory of molecular evolution in bacterial genomes; for example, some genes related to CRISPR are in a constant arms race, which involves a process of coevolution between bacteriophages and bacteria interaction (Takeuchi et al., 2012). In the same way, genes in a COG category like N (including genes related to transfer events between bacterial cells) are under the effect of purifying selection, which involves a tendency to molecular change at the gene level (N’Guessan et al., 2021).

### 3.8. Comparing gene selection pressures among four *Akkermansia* species groups found orthogroups with significantly higher selection among species

Different gene diversities among the main *Akkermansia* species represented in the genome dataset raise the possibility that conserved genes among different species groups have different selective pressures. In order to elucidate that aspect, the pairwise dN/dS ratio was compared from the core, single-copy, orthogroups between a symmetrical amount of genomes (*n* = 19) from those mentioned above the top four *Akkermansia* genomic species. Since dN/dS ratios are metric values for macroevolution, all comparisons were contrasted using the genomic content from *A. glycaniphila* as the outgroup; this ensured that all comparisons were compared with each other using representatives from the same outgroup. Additionally, all dN/dS comparison datasets between representative genomes (having a non-normal distribution, confirmed by the Shapiro–Wilk test) were compared using the Mann–Whitney *U* test, discarding all groups without significant differences.

**Figure 8:**
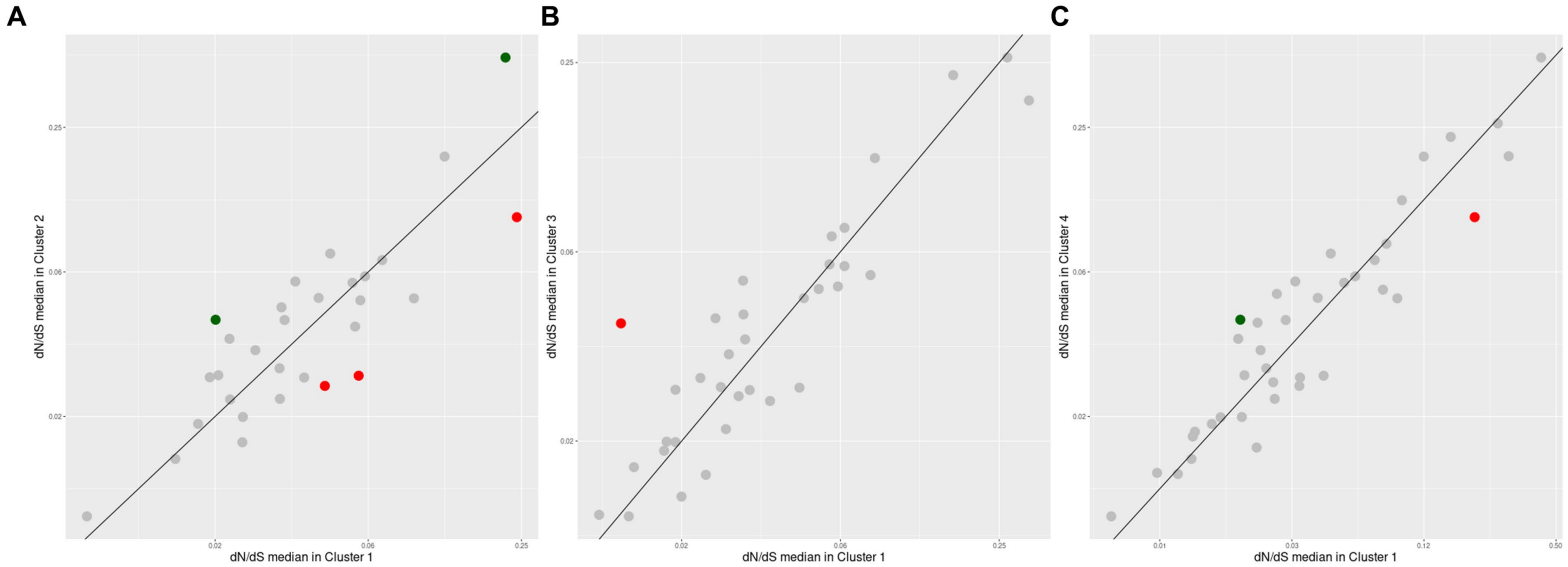
Differential positive selection effects in conserved orthogroups among the top four *Akkermansia* genomic species. Each point is an orthogroup found among two different species which values are compared with the same outgroup (*A. glycaniphila*, type strain). The diagonal line indicates the zone when median pairwise dN/dS in both lineages have the same value. Dots with red or green colors represent orthogroups with remarkably different dN/dS medians (abs(Log2 ratio) > 1, *p* < 0.05, Mann-Whithey *U* test). Comparison corresponds to orthologous from Cluster #1 versus Cluster #2 **(A)**, Cluster #1 versus Cluster #3 **(B)**, and Cluster #1 versus #4 **(C)**.

The comparison between the same set of conserved orthogroups, between two different *Akkermansia* species (Figure 8) shows that despite several orthogroups did not have differential pressure signals, a set of few orthogroups have differentially different evolutionary pressures among species groups. Between Clusters #1 and #2, two orthogroups were found to have a remarkably higher dN/dS median in Cluster #2 against Cluster #1, whereas three orthogroups exhibit the inverse behavior (Figure 8A). When clusters #1 and #3 were compared, only one orthogroup was found exceptionally higher in Cluster #3 (Figure 8B); finally, when clusters #1 and #4 were compared, only one orthogroup was found exceptionally higher in Cluster #4 and one another in Cluster #1 (Figure 8C). The list of the functions represented in those remarked orthogroups were involved in a set of functions such as ribosome assembly and function, energy metabolism, and mucin degradation (Table 2).

**Table 2:** List of orthogroups found with remarkably different dN/dS medians (abs(Log2 ratio) > 1, *p* < 0.05, Mann-Whithey *U* test) across the top four *Akkermansia* species clusters.

Cluster comparison	Feature	OG	Log2 ratio	Orthogroup function
#1 versus #2	Higher in cluster #1	817	1.33	NADH-quinone oxidoreductase subunit B-1
	Higher in cluster #1	863	1.17	NADH-quinone oxidoreductase subunit B-4
	Higher in cluster #2	475	−1.01	Ribosomal protein L16
	Higher in cluster #2	1,357	−1.18	Beta-hexosaminidase
	Higher in cluster #2	1,298	−1.31	NADH-quinone oxidoreductase subunit B-2
#1 versus #3	Higher in cluster #3	1,365	2.01	NADH-quinone oxidoreductase subunit B-3
#1 versus #4	Higher in cluster #1	817	1.12	NADH-quinone oxidoreductase subunit B-1
	Higher in cluster #4	863	−1.01	NADH-quinone oxidoreductase subunit B-4

The composition of a bacterial ribosome is defined as a macromolecule, and due to this different parts could play a specific role in protein synthesis (Lin et al., 2018), in this context, the effects of natural selection could be different depending on the process, either for environmental or antibiotic effects (Byrgazov et al., 2013; Lin et al., 2018). An orthogroup predicted to encode the ribosomal protein L16, was reported with a remarkably positive selection in Cluster #2, in comparison with Cluster #1 (Figure 8A and Table 2). This protein is an important component that helps to stabilize ribosomal structure (Nishimura et al., 2004). Mutations in this protein could be related to the indiscriminate use of antibiotics (Adrian et al., 2000; Gomez et al., 2017), due to the effect these drugs could have on the gut microbiome (Patangia et al., 2022). This study also showed that an orthogroup encoding the NADH-quinone oxidoreductase subunit B (Figures 8B,C and Table 2), which plays a role in energy production and is a multisubunit integral membrane enzyme that participates in different types of respiratory chains (aerobic and anaerobic) and contributes to survival or energy conservation in a variety of lifestyles (Spero et al., 2015). This orthogroup showed a remarkably more positive selection in comparison with clusters #1 versus #3, and #1 versus #4. In this case, this protein could be under positive selection due to the adaptation to environmental changes (Jayaraman et al., 2022).

Interestingly, one of the orthogroups with significantly more positive pressure among species encoded for a beta-hexosaminidase, a protein involved in mucin degradation (Figure 8A and Table 2). As previously mentioned, different members of the gut microbiota interact with the mucus that covers and protects the gastrointestinal epithelium, being capable of degrading glycans that are part of the mucus (Sauvaitre et al., 2021). Mucin is a glycoprotein, and a major component of the mucus layer covering the intestinal epithelium (Becker et al., 2022). Hexosaminidases (members of the GH20 family) is an enzyme that catalyzes the hydrolysis of glycosidic linkages, catalyzing the cleavage of terminal β-D-GlcNAc and β-D-GalNAc residues (Xu et al., 2020), which correspond to a glycoside hydrolases 20 (GH20). Genes encoding this protein were found to have a more positive selection in Cluster #2 versus #1. This effect on selection may reflect the special role of this member of the GH20 in the gut microbiome functional network, where different microbes compete to degrade mucin (Kostopoulos et al., 2021), and this more diversifying selection pressure is a consequence of the need for more variability between different *Akkermansia* lineages to adapt their ability to degrade the carbohydrate moieties of mucin in different contexts, or moreover, to respond to a coevolution process between the glycan structural diversity in the host and glycoside hydrolases (Sonnenburg et al., 2005).

## 4. Discussion

*Akkermansia* is a very interesting group of gut microbiome inhabitants in various vertebrates. This study demonstrated the existence, at the genomic level, of a set of at least 25 species of this genus, including several previously undescribed variants. The presence of several 1-genome clusters suggests the need for the sequencing and discovery of more *Akkermansia* isolates and MAGs. Our evolutionary analysis also showed that the genetic diversity observed through different relevant species clusters through the Tajima D analysis supports the existence of different evolutionary pressures among the member of the genus. The pangenome analysis confirmed that *Akkermansia* species have an open pangenome, suggesting that more gene content diversity remains to be discovered. On the other hand, dN/dS ratio suggests that genes conserved in different lineages show different patterns of natural selection, which can potentially indicate a degree of specialization of different families of proteins that these genes encode, such as the GH20 family, which is related to mucin degradation, and is diversified in the genus.

The use of a gene gain/loss model could show the properties of a hypothetical entity, called *LAkkCA*, that could be the founder member of the genus. A predicted respiratory metabolism, a complete set of carbon metabolism, and a basic set for mucin degradation suggest that *LAkkCA* could be a mucin degrader with microaerobic metabolism, suggesting that this feature is profoundly rooted to the origin of the genus. Despite several studies comparing *Akkermansia* genomes (as previously mentioned), there are only a few studies focused on the evolutionary dynamics of gene gain/loss events [for example (Kim et al., 2022)]. Moreover, no study has been performed considering the underestimated taxonomic diversity of the genus, and no insight into the origin of the genus has been made. Since *Akkermansia* is a genus detected in the GI tract microbiota from mammals, birds, reptiles, or even some amphibians (Zhang et al., 2020), it is reasonable that *Akkermansia* could start to colonize vertebrate GI tract as near as vertebrates appeared. This hypothesis is compatible with the notion that mucins, the main carbon source for described *Akkermansia* species, is a glycoprotein strongly conserved among vertebrates: MUC2, the primary mucin in the mammalian GIT, contained homologs in all vertebrates (Lang et al., 2016). Moreover, it has been proposed that gel-forming mucins could have originated previously from the origin of vertebrates, being found in other metazoans, such as members of Cnidaria, Porifera, and Ctenophora (Lang et al., 2016). Mucins, as important roleplayers in GIT maintenance (Grondin et al., 2020), are important points of contact with microbial activity and metabolism. It is well known that, as a specialized mucin-degrader, *Akkermansia* species are capable of promoting epithelial development in the intestine (Kim et al., 2021) and eliciting, in some conditions, mucin production itself (Shin et al., 2014), among other effects. In the colon, mucins could form a multi-layered structure, with the outer side involved in the interactions with the microbiome (Johansson et al., 2011). Our study showed that, even within the 25 species clusters of *Akkermansia* (comprising a higher taxonomic diversity than previously shown), there is a very conserved gene set involving in mucin degradation and potential interactions between the bacterium and its host, including potential adhesion pathways or special exported proteins mediating surface interactions (such ankyrin proteins) and extracelulluar matrix degradation (such collagenases). These findings support the hypothesis that *Akkermansia* could born inside the GI tract and the ability to degrade mucin could shape its evolution.

In addition, the fact that most of the genes were cataloged in the *Bacteria* domain (through HGT), opens the way to the possibility that the genus *Akkermansia* obtained genes (e.g., GHs) from various intestinal taxa, or they were created by *Akkermansia* spp. and have been transferred to other microorganisms, or even that genes transferred to *Akkermansia* have been perfected through contact with various intestinal microenvironments of different hosts, to be later transferred to different taxa.

Our results show the necessity of new studies with the goal to understand the unique new species and the intraspecific evolutionary processes of *Akkermansia*, as well as, the existence of a copious taxonomic diversity for this genus. A systematic phylogenetic analysis must be implemented to decode the probable taxonomic origin of several HGT-derived genes.

Finally, the need to explore and discover more *Akkermansia* isolates from several other members of the vertebrates may help to gain more insight into the relationship between mucin, the evolution of vertebrates, and the natural history of *Akkermansia*. Moreover, the understanding of the evolutionary process involved in the origin of the machinery to process mucin in *Akkermansia* spp. could help to understand the relationship with the maintenance of gut health, as well as their improvement through the design of probiotics.

## Data availability statement

The original contributions presented in the study are included in the article/Supplementary material, further inquiries can be directed to the corresponding author.

## Author contributions

JC conceived and directed the study. DG, MM-O, BV-V, and JC analyzed the data. All authors collaboratively elaborated, edited, corrected the text and figures in the manuscript, read the manuscript, and approved the content.

## Funding

JC was supported by ANID Fondecyt Project #11200209. BV-V was supported by *ANID Doctorado Nacional*/2021-21211564.

## Conflict of interest

The authors declare that the research was conducted in the absence of any commercial or financial relationships that could be construed as a potential conflict of interest.

## Publisher’s note

All claims expressed in this article are solely those of the authors and do not necessarily represent those of their affiliated organizations, or those of the publisher, the editors and the reviewers. Any product that may be evaluated in this article, or claim that may be made by its manufacturer, is not guaranteed or endorsed by the publisher.

## Footnotes

^1^ https://github.com/SioStef/panplots/

^2^ https://www.ncbi.nlm.nih.gov/research/cog

^3^ https://cran.r-project.org/web/packages/pegas/index.html
