# Supplementary material for: Insights into early evolutionary adaptations of the Akkermansia genus to the vertebrate gut
Source: Front Microbiol. 2023 Sep 14;14:1238580. doi: 10.3389/fmicb.2023.1238580 (PMC10540074; doi:10.3389/fmicb.2023.1238580)
Supplement: Supplementary file 6 [file Presentation_1.pdf]

Supplementary Material

"Insights into early evolutionary adaptations of the *Akkermansia* genus to the vertebrate gut"

1 Supplementary Tables

Supplementary Table 1. List and properties from the final *Akkermansia* selected genome dataset. It includes results from CheckM, GTDB-tk, and the ANI95/MCL clustering (XLSX file).

Supplementary Table 2. Species definition across the *Akkermansia* selected dataset (XLSX file).

Supplementary Table 3. List and annotation data for the reconstructed genes detected in *LAkkCA*. It includes if those genes were newly acquired (XLSX file).

Supplementary Table 4. Content matrix for run_dbCAN output for representative *Akkermansia* genomic species, and the predicted *LAkkCA* and Lineage-A and -B LCA genetic content. Glycosyl hydrolases associated with mucin degradation (see Figure 5) were highlighted (XLSX file).

Supplementary Table 5. Content matrix for MEROPS and SulfAtlas output for representative *Akkermansia* genomic species, and the predicted *LAkkCA* and Lineage-A and -B LCA genetic content (XLSX file).

1.1 Supplementary Figures

Supplementary Figure 1. A detailed version of the *Akkermansia* phylogenomic tree, as is shown in Figure 1.

Supplementary Figure 2. Pangenome accumulation curves to evaluate the openness of the pangenome for each one of the top four *Akkermansia* genomic species clusters with the most members. Clusters I, II,, III and IV are represented by panels A to D, respectively. COG category descriptions are available on the COG Database website (https://www.ncbi.nlm.nih.gov/research/cog).

Supplementary Figure 3. Bar plots representing the relative percentage of counts for COG categories among different pangenome fractions (core, soft-core, shell, cloud, and uniques) in each one of the top four *Akkermansia* genomic species clusters with the most members. Clusters I, II, III, and IV are represented by panels A to D, respectively. COG category descriptions are available on the COG Database website (https://www.ncbi.nlm.nih.gov/research/cog).

Supplementary Figure 4. Detailed gene gain/loss model representing gene content changes across the evolution of the *Akkermansia* genus. The phylogenomic tree from Figure 1 was combined with the orthogroup matrix generated from the Orthofinder output, and analyzed by Count. Black numbers represent the number of shared families for a given ancestor or extant genome; green and red numbers represent gained or missed gene families, respectively. Figure 3 is an abbreviated version of this figure.

Suplementary Figure 5. Boxplots for Tajima D values for core gene sets separated by different COG categories in the top four *Akkermansia* species from Clusters #1 to #4 (A to D, respectively).
