# Supplementary figures and images for: Insights into early evolutionary adaptations of the Akkermansia genus to the vertebrate gut
Source: Front Microbiol. 2023 Sep 14;14:1238580. doi: 10.3389/fmicb.2023.1238580 (PMC10540074; doi:10.3389/fmicb.2023.1238580)

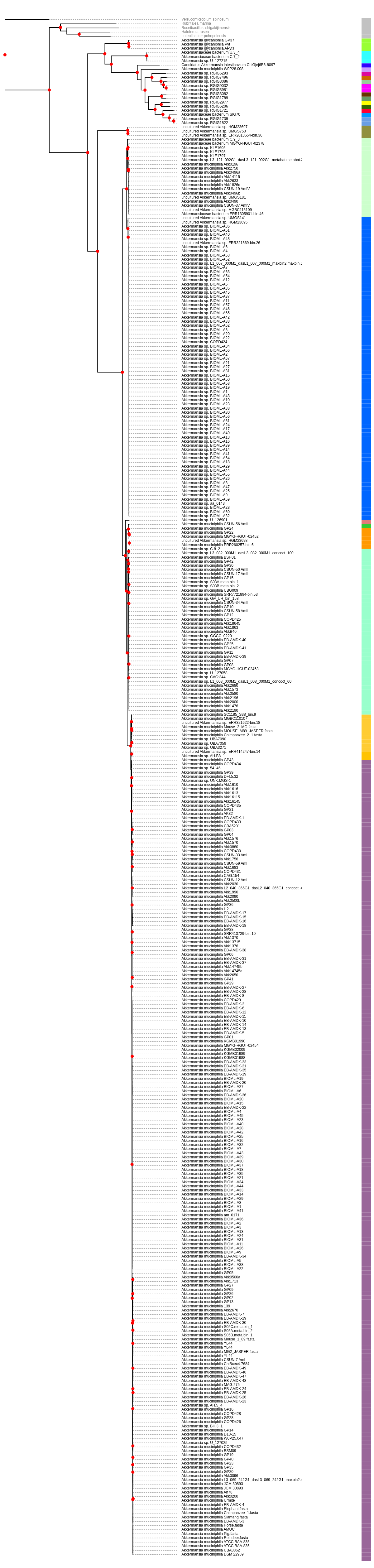

Supplement: Supplementary file 7 [file Image_1.tiff]

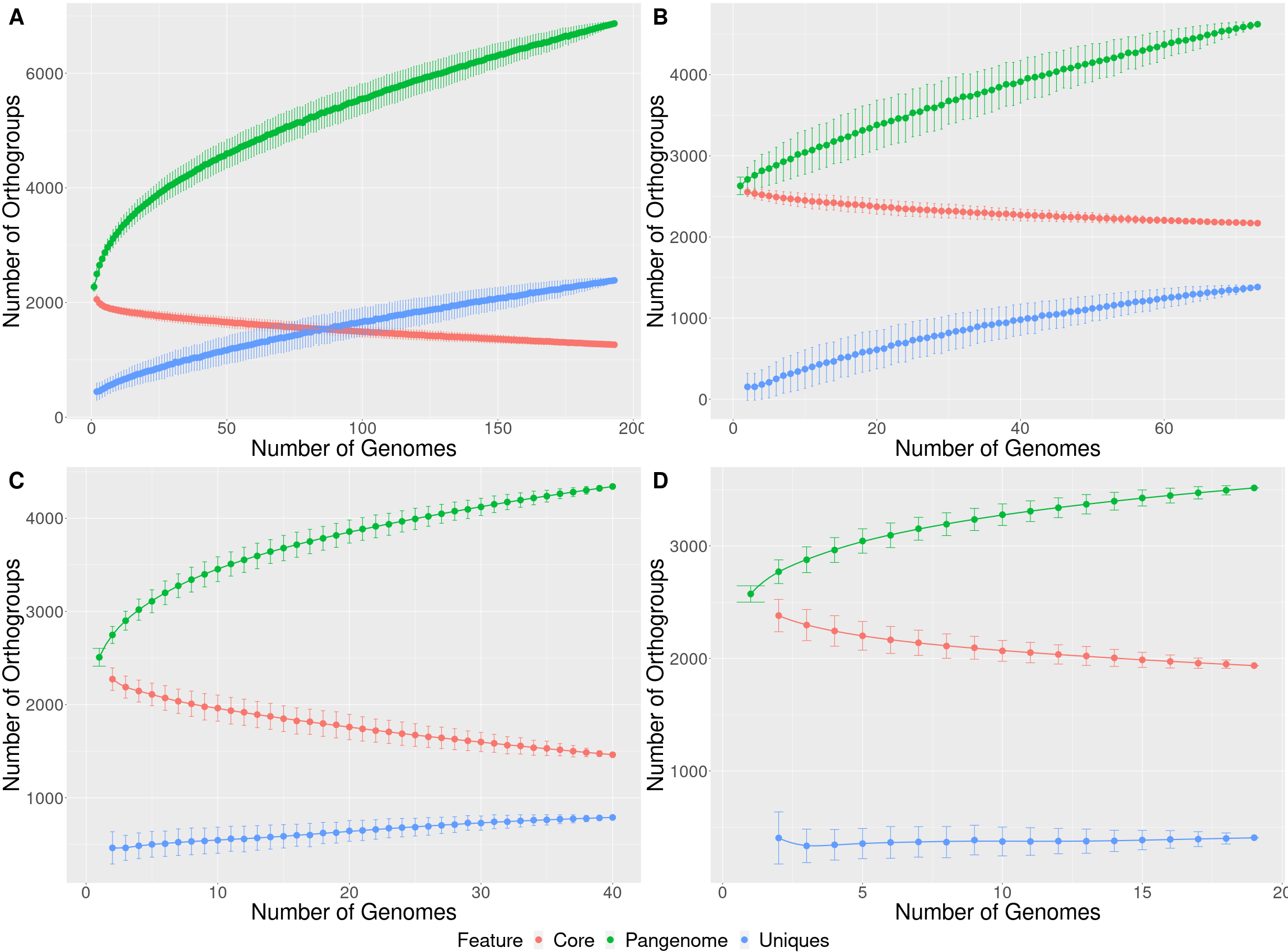

Supplement: Supplementary file 8 [file Image_2.tiff]

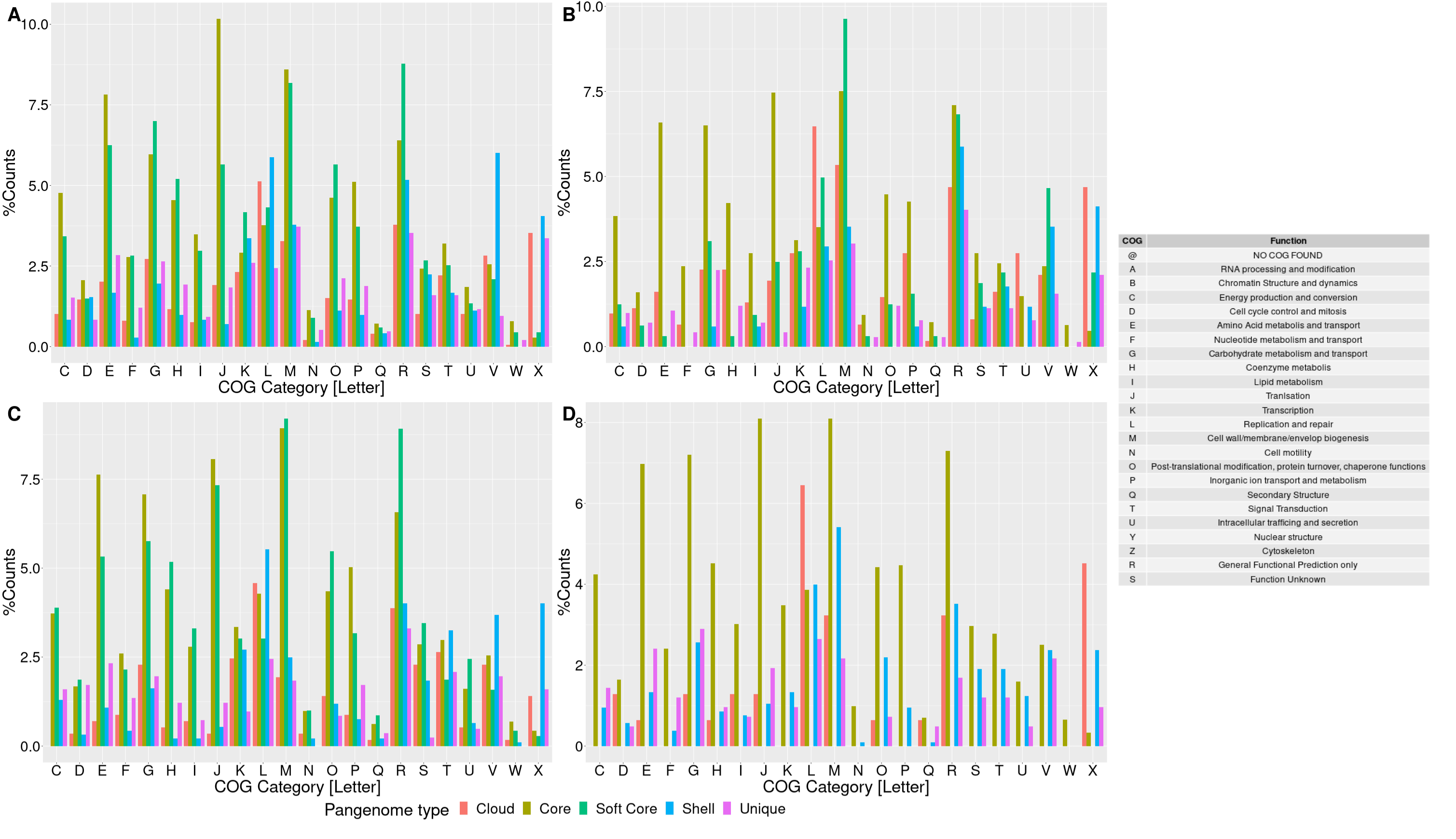

Supplement: Supplementary file 9 [file Image_3.png]

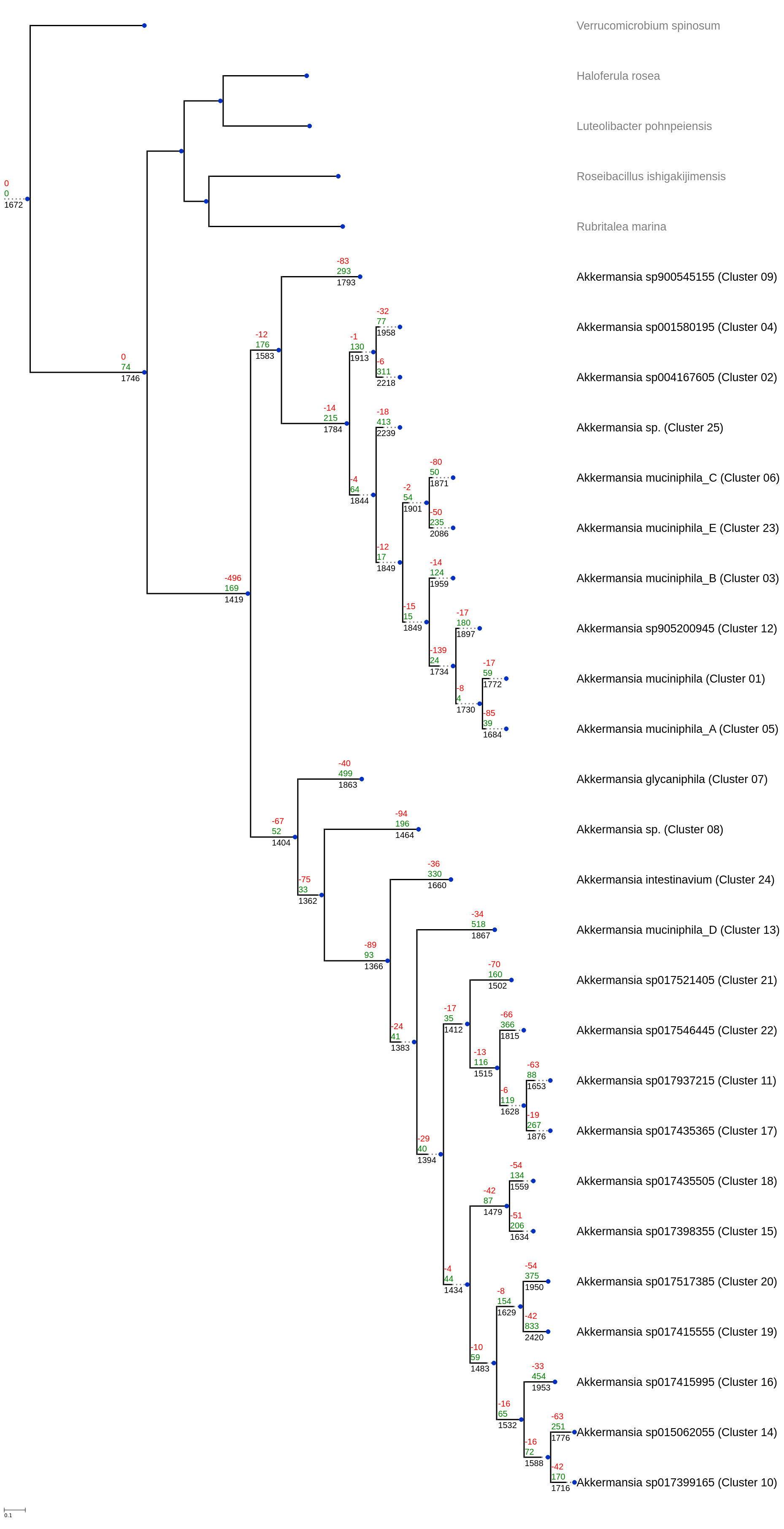

Supplement: Supplementary file 10 [file Image_4.tiff]

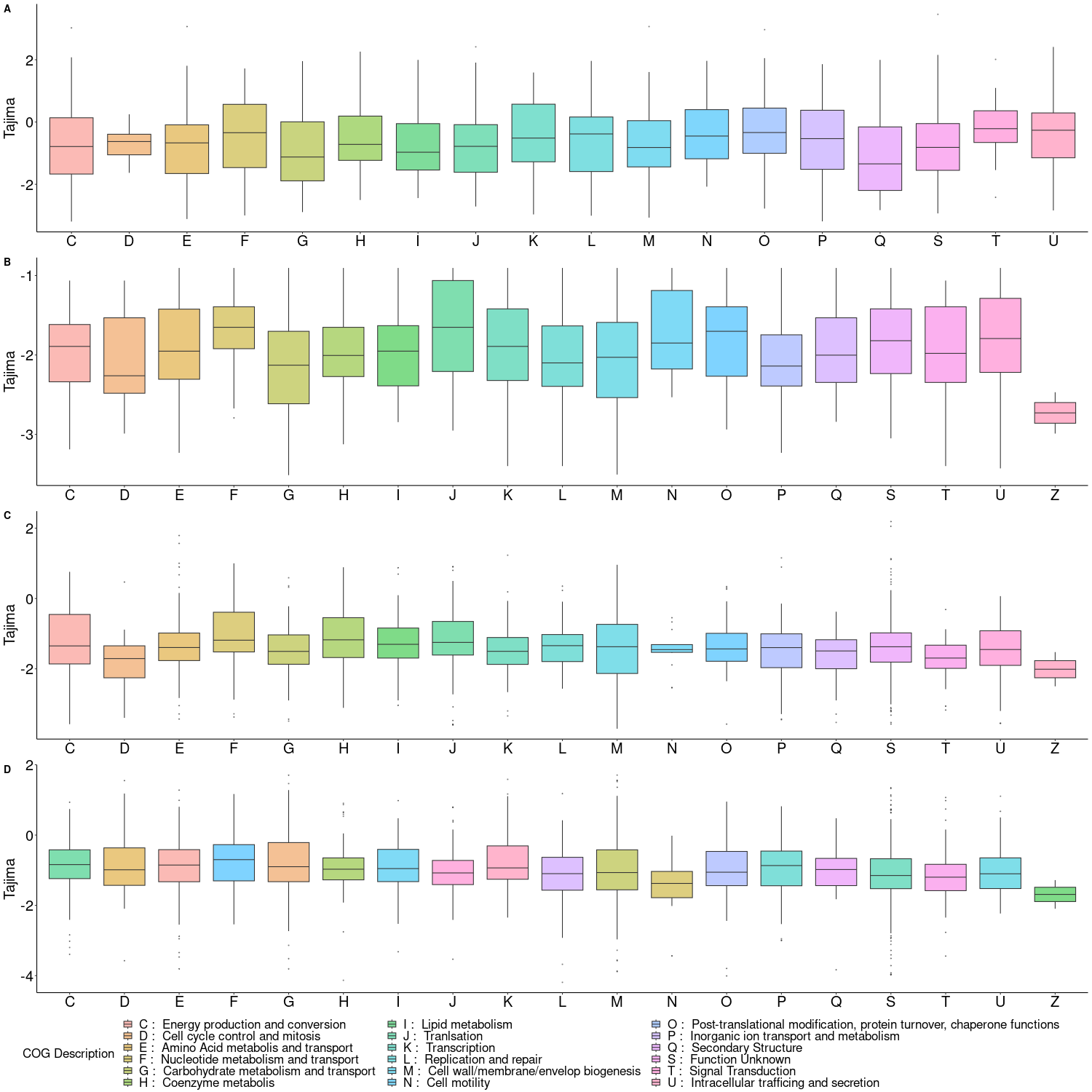

Supplement: Supplementary file 11 [file Image_5.tiff]
